# E3 Ubiquitin Ligase Cbl-b Suppresses Proallergic T Cell Development and Allergic Airway Inflammation

**DOI:** 10.1016/j.celrep.2014.01.012

**Published:** 2014-02-06

**Authors:** Guilin Qiao, Haiyan Ying, Yixia Zhao, Yanran Liang, Hui Guo, Huifeng Shen, Zhenping Li, Julian Solway, Enxiang Tao, Y. Jeffrey Chiang, Stanley Lipkowitz, Josef M. Penninger, Wallace Y. Langdon, Jian Zhang

**Affiliations:** 1 Section of Nephrology, Department of Medicine, University of Chicago, Chicago, IL 60637, USA; 2 Section of Pulmonary and Critical Care, Department of Medicine, University of Chicago, Chicago, IL 60637, USA; 3 Committee on Immunology, University of Chicago, Chicago, IL 60637, USA; 4 Committee on Molecular Pathogenesis and Molecular Medicine, University of Chicago, Chicago, IL 60637, USA; 5 Department of Microbial Infection and Immunity, Ohio State University, Columbus, OH 43210, USA; 6 Experimental Immunology Branch, Center for Cancer Research, National Cancer Institute, National Institutes of Health, Bethesda, MD 20892, USA; 7 Women’s Malignancies Branch, Center for Cancer Research, National Cancer Institute, National Institutes of Health, Bethesda, MD 20892, USA; 8 Institute of Molecular Biotechnology, Austrian Academy of Sciences, 1030 Vienna, Austria; 9 School of Pathology and Laboratory Medicine, University of Western Australia, Crawley, WA 6009, Australia; 10 Department of Cardiology, Xiangya Hospital, Central South University, Changsha, 410008, China; 11 Department of Neurology, Sun Yat-Sen Memorial Hospital of Sun Yat-Sen University, Guangzhou, 510120, China

## Abstract

E3 ubiquitin ligase Cbl-b has emerged as a gatekeeper that controls the activation threshold of the T cell antigen receptor and maintains the balance between tolerance and autoimmunity. Here, we report that the loss of Cbl-b facilitates T helper 2 (Th2) and Th9 cell differentiation in vitro. In a mouse model of asthma, the absence of Cbl-b results in severe airway inflammation and stronger Th2 and Th9 responses. Mechanistically, Cbl-b selectively associates with Stat6 upon IL-4 ligation and targets Stat6 for ubiquitination and degradation. These processes are heightened in the presence of T cell receptor (TCR)/ CD28 costimulation. Furthermore, we identify K108 and K398 as Stat6 ubiquitination sites. Intriguingly, introducing Stat6 deficiency into Cblb^−/−^ mice abrogates hyper-Th2 responses but only partially attenuates Th9 responses. Therefore, our data reveal a function for Cbl-b in the regulation of Th2 and Th9 cell differentiation.

## INTRODUCTION

Antigenic stimulation of T cells drives naive CD4 T helper (Th) cells into functionally distinct subsets of Th cells that are dependent upon many factors, including the affinity of T cell receptor (TCR) for antigen, the concentration of antigen during TCR triggering, and in particular the cytokine milieu (Murphy et al., 2000). Th1 cells are characterized by the production of proinflammatory interferon γ (IFN-γ) to mediate cellular immunity, whereas Th2 cells produce interleukin-4 (IL-4), IL-5, and IL-13, and are responsible for regulating humoral immunity and, in pathological conditions, asthma and allergy. Recently, newly identified Th17 cells, distinct from Th1 and Th2 cells, were shown to produce IL-17, IL-17F, IL-22, and IL-21, and mediate tissue inflammation (Harrington et al., 2006; Park et al., 2005). In addition to Th1, Th2, and Th17, a recently identified Th subset specialized for the production of IL-9, termed Th9, was shown to be generated in the presence of transforming growth factor β (TGF-β) and IL-4 (Dardalhon et al., 2008; Veldhoen et al., 2008). Th9 cells are related to Th2 cells in that they require signal transducer and activator of transcription 6 (Stat6), GATA-binding protein 3 (GATA3), and interferon-regulatory factor 4 (IRF4) for development but are distinct from Th2 cells in their requirement for PU.1 (Chang et al., 2010; Kaplan, 2013; Staudt et al., 2010). Th9 cells have also been shown to contribute to allergic inflammation (Chang et al., 2010; Kaplan, 2013; Yao et al., 2013).

IL-4 is the determining factor for Th2 cell differentiation. In this regard, IL-4 is a key cytokine in the development of allergic inflammation (Chatila, 2004). Binding of IL-4 to the IL-4 receptor (IL-4R) triggers phosphorylation of Janus kinase-1 (JAK-1) and JAK-3, leading to the activation of Stat6. Tyrosine-phosphorylated Stat6 forms homodimers and translocates into the nucleus, where it binds IL-4-responsive elements (Takeda et al., 1996; Wurster et al., 2000), which, together with NF-AT, AP-1, NF-κB, and other TCR-induced signal mediators, activates the transcription of IL-4 as well as the transcription factor GATA3, a signature mediator of Th2 lineage commitment. Stat6 has also been documented to be critical for induction of Th9 cell differentiation (Goswami et al., 2012). However, the molecular basis of how the signals derived from TCR and IL-4R can be integrated has yet to be defined.

Cbl-b is an E3 ubiquitin ligase that contains multiple domains, including a protein tyrosine kinase-binding (TKB) domain, a RING-finger (RF) domain, and a proline-rich region. The RF domain is the site in which Cbl family proteins recruit ubiquitin-conjugating enzymes, which add ubiquitin to targeted proteins. The TKB domain has been shown to recognize specific phosphotyrosine residues on target proteins for ubiquitin conjugation (Thien and Langdon, 2005). These domains are required for Cbl proteins to regulate cell signaling and protein degradation. Gene targeting in mice has indicated that Cbl-b is a gatekeeper that maintains a balance between immunity and tolerance. Indeed, signaling via CD28 and CTLA-4 tightly regulates Cbl-b expression (Zhang et al., 2002; Li et al., 2004), which is critical for establishing the threshold for T cell activation and tolerance. In strong support of this notion, *Cblb*^−/−^ T cells are resistant to anergy induction in vitro and in vivo (Heissmeyer et al., 2004; Jeon et al., 2004). However, whether Cbl-b also plays a role in Th cell differentiation remains to be elucidated.

In this study, we report that loss of Cbl-b leads to a skewed Th2 and Th9 phenotype, which results in augmented airway inflammation. Upon IL-4 stimulation, Cbl-b specifically associates with Stat6, targeting it for ubiquitination and degradation, a process that is further heightened by TCR signaling. Surprisingly, Stat6 deficiency abrogates hyper-Th2 responses but only partially attenuates Th9 responses in *Cblb*^−/−^ mice. This suggests that Cbl-b regulates Th2 cell differentiation via a Stat6-dependent mechanism but regulates Th9 cell differentiation via both Stat6-dependent and -independent mechanisms.

## RESULTS

### Cbl-b Negatively Regulates Th2 and Th9 Cell Differentiation In Vitro

To assess whether Cbl-b affects Th cell differentiation, we measured the expression of Cbl-b protein in differentiated Th1, Th2, and Th17 cells by intracellular staining. Although the expression of Cbl-b was increased in Th1, Th2, and Th17 cells, as determined by the mean fluorescence intensity (MFI) of Cbl-b, its expression was significantly lower in Th2 cells than in Th1 and Th17 cells, suggesting that Cbl-b may play a role in Th2 cell differentiation (Figure S1A). The increase in Cbl-b protein expression upon chronic TCR stimulation is consistent with our previous report (Li et al., 2004). To confirm this further, we assessed the expression of Cbl-b in differentiating Th1, Th2, Th9, and Th17 cells by intracellular staining. Cbl-b expression was markedly lower in Th9 and Th2 cells than in Th1 and Th17 cells during the differentiation process, with the lowest levels occurring in Th9 cells (Figure S1B).

**Figure 1:**
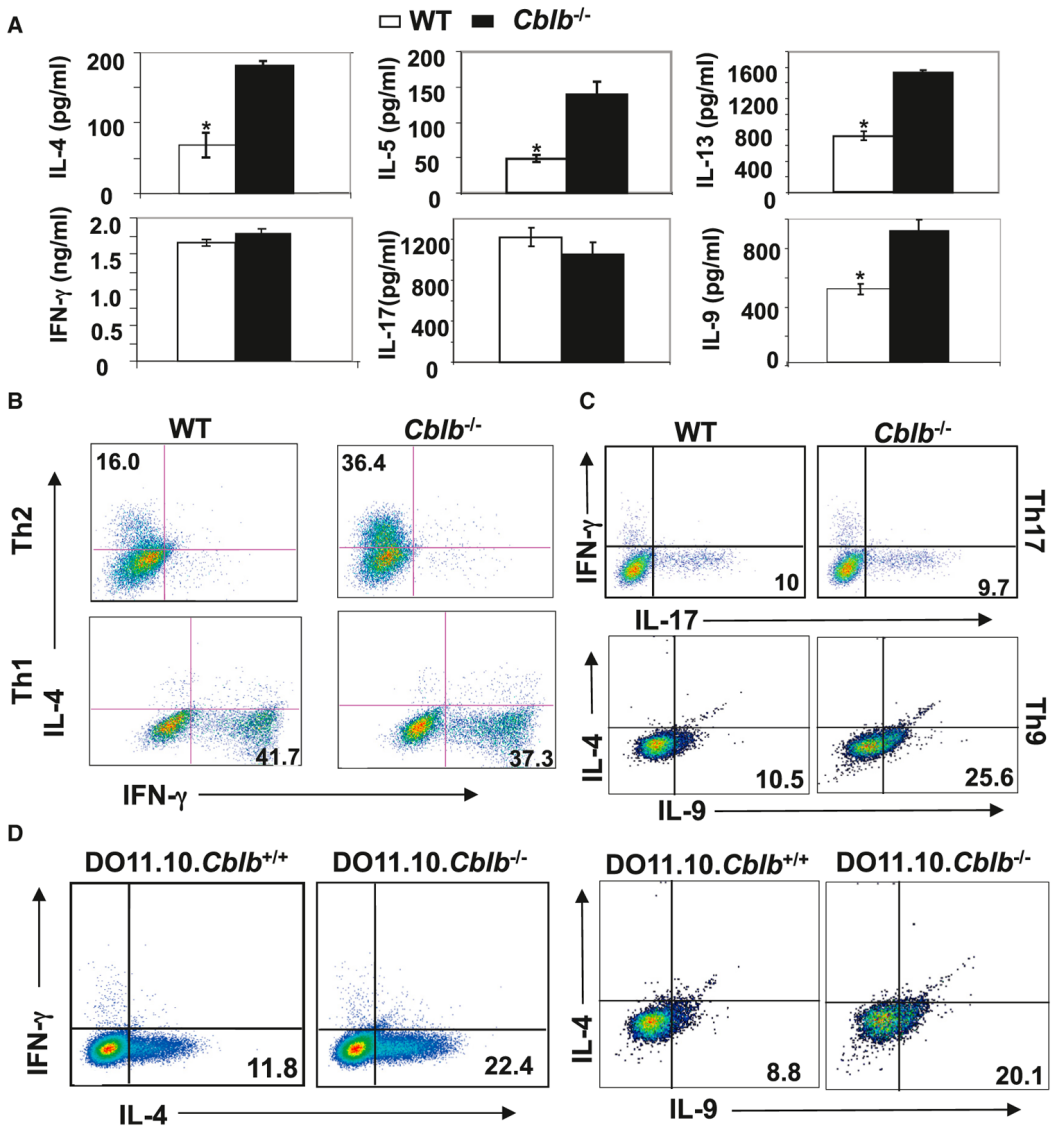
Loss of Cbl-b Favors Th2 and Th9 Cell Differentiation In Vitro (A) ELISA results for cytokine production of purified naive CD4^+^CD25^−^CD62L^hi^CD44^lo^ T cells from WT or *Cblb*^−/−^ mice upon stimulation with plate-bound anti-CD3 plus anti-CD28 for 48 hr (*p < 0.05, compared with *Cblb*^−/−^ mice). (B and C) Intracellular staining of Th1, Th2, Th9, and Th17 cells differentiated in vitro from purified naive CD4^+^CD25^−^CD62L^hi^CD44^lo^ T cells of WT or *Cblb*^−/−^ mice. Numbers in the quadrants in (B) indicate the percentage of IL-4/IFN-γ-producing cells in the CD4^+^ population. Numbers in the quadrants in (C) indicate the percentage of IL-17/IL-9-producing cells in the CD4^+^ population. (D) Intracellular staining of Th2 cells and Th9 cells differentiated in vitro from DO11.10 and DO11.10.*Cblb*^−/−^ naive CD4^+^ cells. Numbers in the quadrants indicate the percentage of IL-4- and IL-9-producing cells. Data are representative of three independent experiments.

To determine the potential role of Cbl-b in Th cell differentiation, we examined the cytokine profile of naive CD4^+^ T cells from wild-type (WT) and *Cblb*^−/−^ mice upon CD3/CD28 stimulation. Although WT and *Cblb*^−/−^ T cells produced comparable levels of IFN-γ and IL-17, production of IL-4, IL-5, IL-9, and IL-13 was significantly higher in *Cblb*^−/−^ T cells than in WT T cells (Figure 1A). The increased production of IL-4, IL-5, IL-9, and IL-13 by *Cblb*^−/−^ T cells supports the notion that Cbl-b may inhibit Th2 and Th9 cytokine production.

To further analyze a possible functional role for Cbl-b in Th2 and Th9 cell differentiation, we performed in vitro Th1, Th2, Th9, and Th17 differentiation assays. We observed an augmented generation of IL-4+ Th2 cells and IL-9^+^ Th9 cells derived from naive *Cblb*^−/−^ CD4^+^ T cells in comparison to those derived from naive WT CD4^+^ T cells (Figure 1B). However, when cells were cultured under Th1 and Th17 polarizing conditions, no difference was observed in the generation of IFN-γ^+^ Th1 cells or IL-17^+^ Th17 cells from WT and *Cblb*^−/−^ T cells (Figures 1B and 1C). To further confirm this observation using homogeneous populations of naive CD4^+^ T cells, we generated DO11.10.*Cblb*^+/+^ and DO11.10.*Cblb*^−/−^ mice in which the I-A^d^-restricted DO11.10 TCR transgene is specific for a peptide from chicken ovalbumin (OVA; aa 323–339, OVAp_323–339_) (Murphy et al., 1990). Consistent with the data shown in Figure 1B, loss of Cbl-b led to biased Th2 and Th9 cell differentiation of DO11.10 T cells (Figure 1D).

### Cblb^−/−^ Mice Are Highly Susceptible to Asthma Induction and Display Heightened Th2 and Th9 Responses

**Figure 2:**
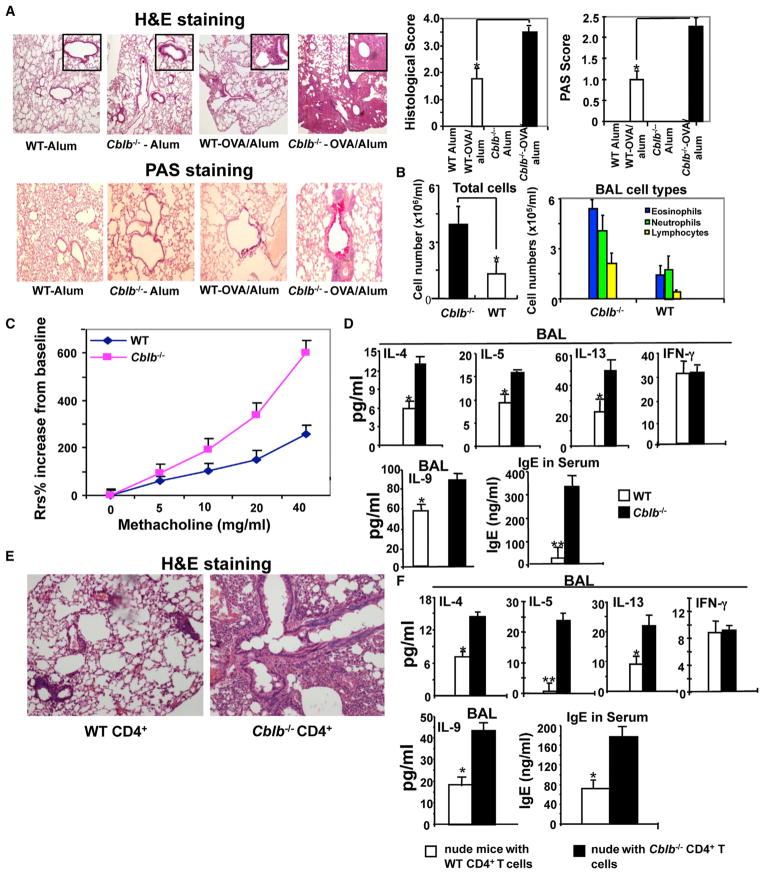
*Cblb*^−/−^ Mice Are Highly Susceptible to Asthma Induction and Display Heightened Th2 and Th9 Responses (A) Airway inflammation and mucus production in OVA-sensitized WT and *Cblb*^−/−^ mice as determined by H&E staining (top) and PAS staining (bottom). Original magnification, ×40 (H&E), ×100 (PAS). Semiquantitative analysis of the severity of peribronchial inflammation and the abundance of PAS-positive mucus-containing cells was performed (n = 5; *p < 0.05, compared with *Cblb*^−/−^ mice). (B) Inflammatory cells from BAL fluid. (C) Respiratory system resistance (Rsr) in WT and *Cblb*^−/−^ mice after OVA rechallenge. (D) Serum IgE and BAL IFN-γ, IL-4, IL-5, IL-9, and IL-13 detected by ELISA (*p < 0.05 and **p < 0.01, compared with *Cblb*^−/−^ mice). (E) H&E staining of lungs in BALB/c nude mice (n = 4) that were adoptively transferred (i.v.) with naive CD4^+^ T cells (5 ×10^6^) from WT or *Cblb*^−/−^ mice, permitted to equilibrate 30 days to avoid homeostatic proliferation, and immunized with OVA as in (A). Original magnification ×100. (F) IFN-γ, IL-4, IL-5, IL-9, and IL-13 concentrations in the BAL fluid and IgE in the serum of OVA-sensitized BALB/c nude mice receiving naive WT or *Cblb*^−/−^ CD4^+^ T cells, detected by ELISA (*p < 0.05 and **p < 0.01, compared with *Cblb*^−/−^ mice). Data represent three independent experiments (mean ± SD).

To determine whether Cbl-b regulates Th2 and Th9 responses in vivo, we used a mouse model of allergic asthma, which has been shown to be mediated by both Th2 and Th9 cytokines (Kaplan, 2013). As shown in Figure 2A, after immunization and challenge with OVA, *Cblb*^−/−^ mice displayed more severe inflammatory cell infiltration in the perivascular and peribronchial areas, goblet cell metaplasia, and increased mucus production, as determined by hematoxylin and eosin (H&E) and periodic acid-Schiff (PAS) staining of lung sections. As expected, *Cblb*^−/−^ mice had significantly more infiltrating cells and eosinophils in bronchoalveolar lavage (BAL) fluid than WT mice (Figure 2B).

Airway hyperresponsiveness (AHR) is a hallmark of asthma and is often associated with increased airway inflammation (Pernis and Rothman, 2002). To investigate whether the severe airway inflammation in *Cblb*^−/−^ mice also leads to heightened AHR, we measured respiratory system resistance (Rsr) changes in response to methacholine (MetCh) aerosol at 24 hr after the final dose of OVA challenge as described previously (Myou et al., 2003). We found that *Cblb*^−/−^ mice remained hyperresponsive to MetCh (Figure 2C). Cytokine analysis showed that Cbl-b^−/−^ mice had significantly higher levels of IL-4, IL-5, IL-13, and IL-9 in BAL fluid compared with WT mice, which closely correlated with serum immunoglobulin E (IgE) production (Figure 2D). Note that the IFN-γ level in the BAL of *Cblb*^−/−^ mice was comparable to that of WT mice (Figure 2D). These data indicate that the loss of Cbl-b specifically inhibits Th2 and Th9 responses in vivo.

As Cbl-b deficiency has been shown to affect B cells, monocytes, and mast cells (Bachmaier et al., 2007; Qiao et al., 2007; Gustin et al., 2006), which may potentially affect Th2 cell differentiation, we investigated whether T cell-intrinsic loss of Cbl-b results in heightened airway inflammation and aberrant Th2 responses. We therefore analyzed BALB/c nude mice that were reconstituted with WT or *Cblb*^−/−^ naive CD4^+^CD25^−^ T cells. As shown in Figure 2E, BALB/c nude mice that received naive *Cblb*^−/−^ CD4^+^ T cells displayed more severe airway inflammation as well as heightened IL-4, IL-5, IL-9, and IL-13 in the BAL fluid, and IgE in the serum, than those that received naive WT CD4^+^ T cells (Figure 2F). These findings indicate that Cbl-b deficiency in T cells is sufficient for stronger Th2 and Th9 responses in vivo.

As loss of Cbl-b lowers the threshold for T cell activation (Bachmaier et al., 2000; Chiang et al., 2000; Guo et al., 2012), one would expect that the heightened Th2 and Th9 responses in *Cblb*^−/−^ mice might result from hyperresponsiveness to antigen stimulation. To test this, we adoptively transferred naive CD4^+^ T cells from DO11.10.*Rag1*^−/−^ and DO11.10.*Rag1*^−/−^ *Cblb*^−/−^ mice into WT BALB/c recipients, and then immunized with OVA at 100 μg/ml in alum, which is the dose used to induce asthma. We monitored the expression of early T cell activation markers, including CD25 and CD69, in antigen-specific KJ-1-26^+^ cells during T cell activation. Our data show that immunization with OVA peptide at a 100 μg/ml in alum did not induce heightened levels of activation in *Cblb*^−/−^ T cells, as revealed by the surface expression of the early activation markers CD25 and CD69 (Figure S2). Our data therefore suggest that the heightened Th2 and Th9 responses and allergic airway inflammation observed in the absence of Cbl-b are not likely due to hyperresponsiveness of Th2 or Th9 cells to antigen stimulation in vivo. This is further supported by the fact that comparable amounts of IFN-γ were detected in the BAL fluid of OVA/alum-immunized WT and *Cblb*^−/−^ mice or BALB/c nude mice that received naive CD4^+^ T cells from WT and *Cblb*^−/−^ mice (Figures 2D and 2F), because the lower threshold should also lead to enhanced IFN-γ in the BAL fluid.

### Cbl-b Selectively Inhibits IL-4/Stat6 Signaling in T Cells

We next sought to determine the molecular mechanism by which Cbl-b inhibits Th2 and Th9 cell differentiation. It has been well documented that signals derived from both the TCR and IL-4R are required for Th2 or Th9 cell differentiation (Wan and Flavell, 2009; Kaplan, 2013). It has been shown that Cbl-b does not regulate TGF-β signaling in T cells (Harada et al., 2010; Qiao et al., 2013). We previously showed that TCR/CD28-induced activation of MAPKs, NF-κB, and NF-AT is comparable between WT and *Cblb*^−/−^ T cells (Qiao et al., 2008), suggesting that the potentiation of Th2 and Th9 cell differentiation in the absence of Cbl-b may be due to augmented expression of IL-4R or aberrant activation of IL-4R signaling. The expression of IL-4Rα was comparable between WT and *Cblb*^−/−^ CD4^+^ T cells (data not shown), suggesting that the heightened Th2 and Th9 cell differentiation in *Cblb*^−/−^ T cells does not result from increased IL-4Rα expression.

**Figure 3:**
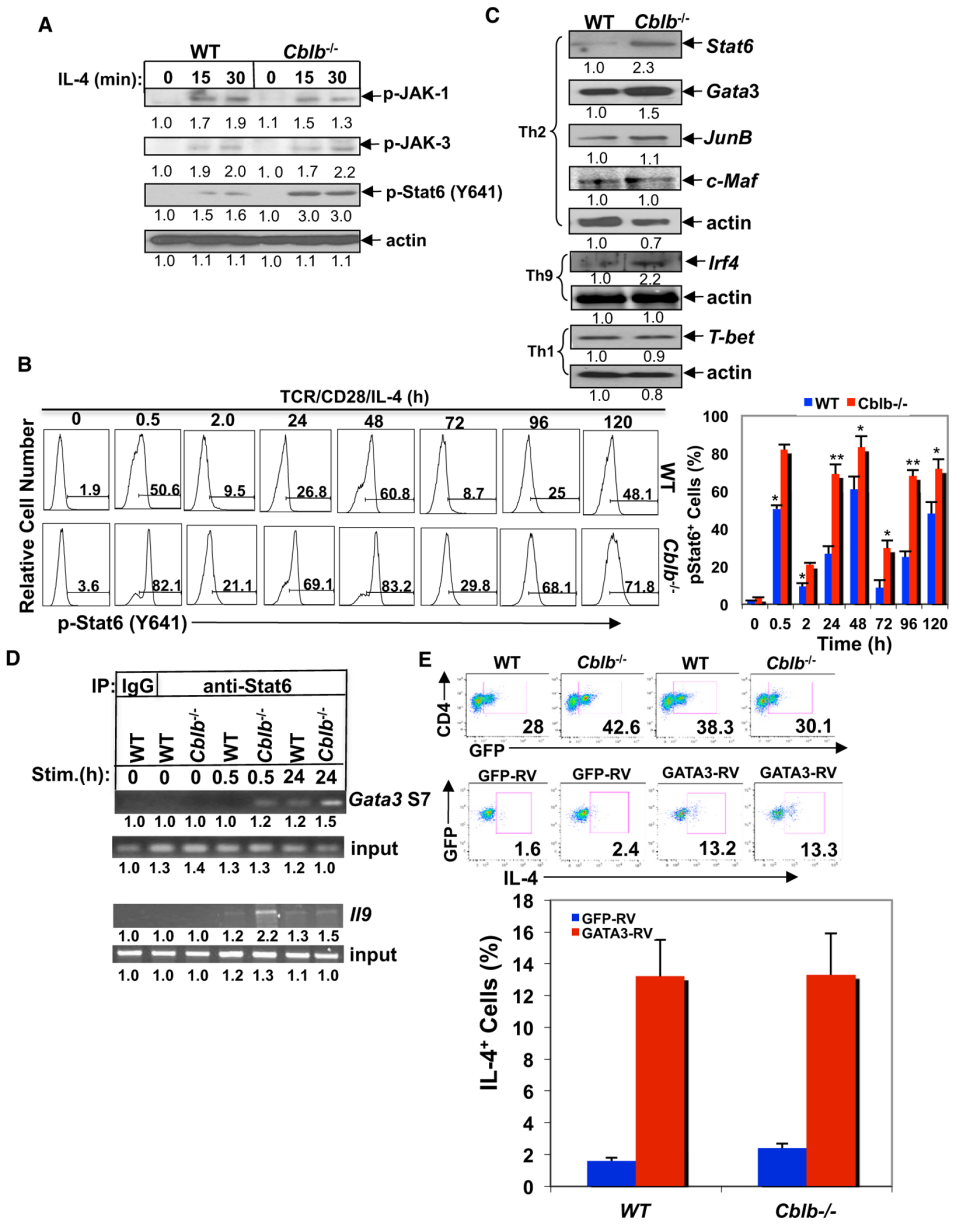
Loss of Cbl-b Results in Heightened Activation of Stat6 (A) Immunoassay of phosphorylation of JAK-1, JAK-3, and Stat6 in CD4^+^ T cells of WT and *Cblb*^−/−^ mice treated with mouse IL-4 (5 ng/ml) at different times. (B) Flow-cytometric analysis of the phosphorylation of Stat6 at Y641 in WT and *Cblb*^−/−^ CD4^+^ T cells during Th2 cell differentiation. (C) Immunoassay of transcription factors (Stat6, GATA3, c-Maf, JunB, IRF4, and T-bet) in nuclear extracts of differentiated Th1, Th2, and Th9 cells from WT and *Cblb*^−/−^ mice in response to anti-CD3 restimulation for 4 hr. β-actin was used to indicate equal protein loading. (D) ChIP assay of Stat6 binding to the *Gata3* S7 region or the *Il9* promoter region in nuclear extracts of naive WT and *Cblb*^−/−^CD4^+^ T cells stimulated with anti-CD3, anti-CD28, and IL-4, or anti-CD3, anti-CD28, IL-4, and TGF-β for 0.5 hr and 24 hr. The band intensities were quantified using the Li-Cor Odyssey Imaging System. (E) Naive CD4^+^ T cells from WT and *Cblb*^−/−^ mice cultured under Th2 differentiation conditions and retrovirally transfected with two bicistronic retroviruses expressing GFP-GATA3 or GFP vector. The IL-4-expressing cells were assessed by intracellular staining. Data shown are gated on the GFP^+^ population. Data are representative of three independent experiments.

Triggering of IL-4R by IL-4 induces activation of JAKs/Stat6 signaling and is essential for initiation of both Th2 and Th9 cell differentiation (Kaplan et al., 1996; Kaplan, 2013). We therefore assessed the phosphorylation of JAKs/Stat6 in response to IL-4 in WT and *Cblb*^−/−^ CD4^+^ T cells. Although JAK-1 and JAK-3 phosphorylation was comparable between WT and *Cblb*^−/−^ CD4^+^ T cells, IL-4-induced Stat6 phosphorylation at Y641 was much stronger in *Cblb*^−/−^ CD4^+^ T cells than in WT CD4^+^ T cells (Figure 3A). To assess the state of Stat6 Y641 phosphorylation during Th2 cell differentiation, we measured Stat6 Y641 phosphorylation. We found that Stat6 Y641 phosphorylation was induced in both WT and *Cblb*^−/−^ T cells during Th2 cell differentiation, and that the loss of Cbl-b led to a greater increase of Stat6 Y641 phosphorylation at all time points (Figure 3B). The kinetics of Stat6 phosphorylation revealed a first peak at 0.5 hr after stimulation, a second peak at day 2, a decline at day 3, and another increase at days 4 and 5. Our data suggest that the first peak of Stat6 phosphorylation at Y641 may represent the direct effect of exogenous IL-4 added in the culture, whereas the second and third peaks may be due to the endogenous secretion of IL-4 in the culture.

Since Stat6 is important for the induction of GATA3 (Murphy et al., 2000), we measured the nuclear expression of Stat6, GATA3, and other transcription factors involved in Th2 cell differentiation, and IRF4 from differentiated Th9 cells. We found that expression of Stat6, GATA3, and IRF4 was increased in *Cblb*^−/−^ CD4^+^ T cells (Figure 3C). In contrast, the nuclear expression of T-bet, the master transcription factor for Th1 cell differentiation (Szabo et al., 2000; Murphy et al., 2000), was comparable between WT and *Cblb*^−/−^ CD4^+^ T cells under Th1-biased conditions (Figure 3C), consistent with the comparable Th1 cell differentiation in vitro between WT and *Cblb*^−/−^ T cells, as shown in Figure 1B. We also did not observe an increase in PU.1 expression by differentiated *Cblb*^−/−^ Th9 cells (data not shown), suggesting a more important role for IRF4 in Th9 differentiation in the absence of Cbl-b.

The binding sites of Stat6 at the *Gata3* gene locus and *Il9* promoter were recently identified (Onodera et al., 2010; Yang et al., 2013); therefore, we tested whether the absence of Cbl-b results in increased binding of Stat6 at the *Gata3* and *Il9* promoter region by performing Stat6 chromatin immunoprecipitation (ChIP) assays using *Gata3* and *Il9* as the target genes. We found markedly augmented Stat6 binding to the *gata3* S7 region in CD4^+^ T cells lacking Cbl-b at 30 min and 24 hr of stimulation with TCR/CD28 and IL-4, or increased binding of Stat6 to the *Il9* promoter region in *Cblb*^−/−^ CD4^+^ T cells at 30 min of stimulation with TCR/CD28, IL-4, and TGF-β (Figure 3D).

To further verify that Cbl-b negatively regulates GATA3 via Stat6, WT and *Cblb*^−/−^ CD4^+^ T cells were retrovirally infected with GATA3 and stimulated with anti-CD3 and anti-CD28 in the presence of anti-iL-4, which blocks IL-4/Stat6 signaling. Overexpression of GATA3 in both WT and *Cblb*^−/−^ T cells bypassed the IL-4/Stat6 signaling to equally drive Th2 cell differentiation (Figure 3E), further indicating that Cbl-b suppresses Th2 cell differentiation upstream of GATA3.

### Cbl-b Physically Associates with Stat6 upon IL-4 or TCR/CD28 Stimulation

**Figure 4:**
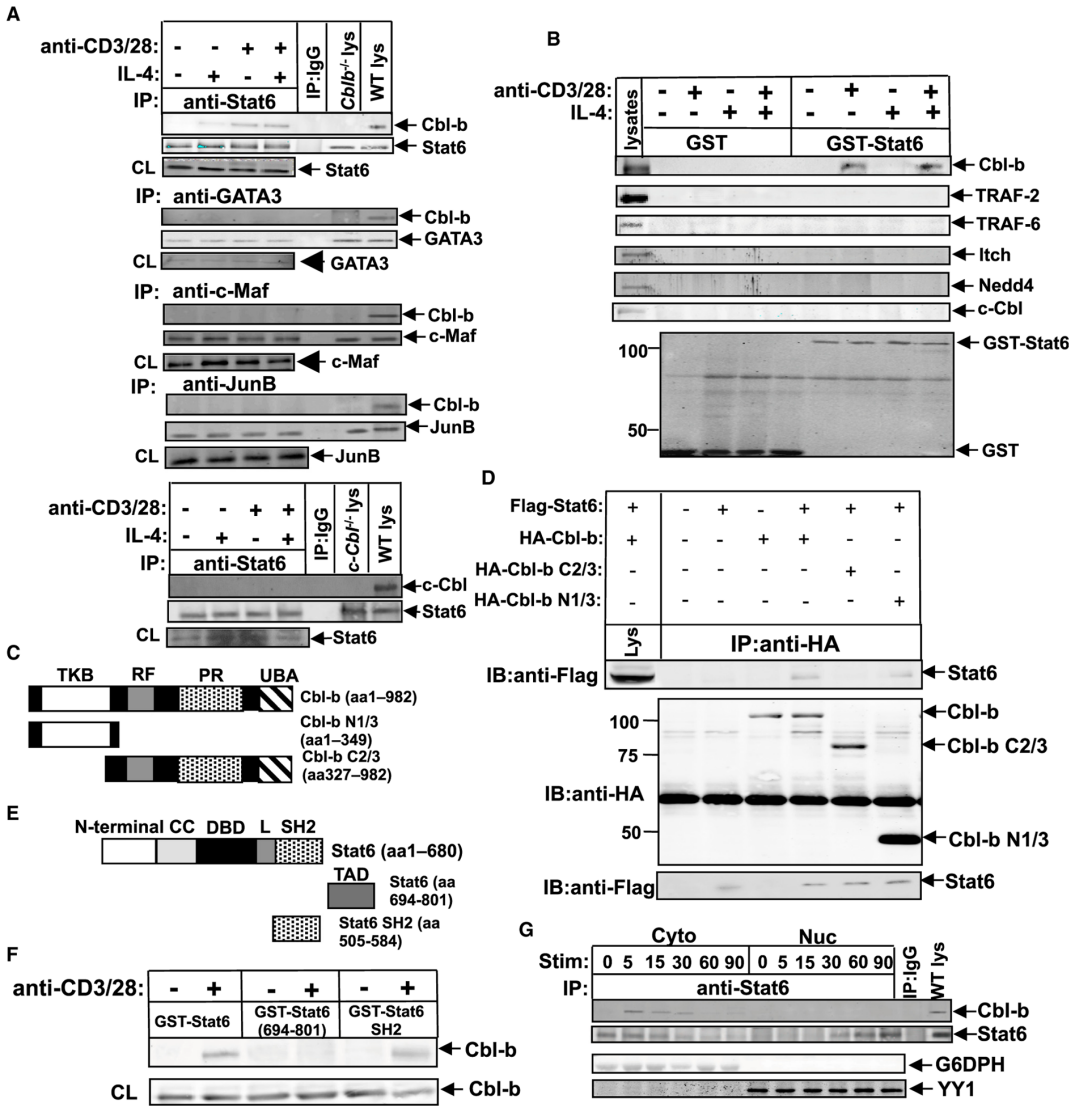
Stat6 Specifically Associates with Cbl-b upon IL-4 or TCR/CD28 Stimulation (A) Immunoprecipitation (IP) of proteins from CD4^+^ T cells purified from BALB/c mice treated with mouse IL-4 (5 ng/ml), anti-CD3 plus anti-CD28, or both for 15 min with anti-Stat6, anti-GATA-3, anti-c-Maf, and anti-JunB, respectively, and blotted with anti-Cbl-b or anti-c-Cbl. (B) Affinity precipitation of lysates from CD4^+^ T cells stimulated with IL-4 or anti-CD3 plus anti-CD28, or both with GST or GST-Stat6 (aa 1–680), captured by glutathione sepharose beads, and analyzed by immunoblot analysis with antibodies against Cbl-b, Itch, TRAF-2, TRAF-6, c-Cbl, and Nedd4. The expression of GST fusion protein was confirmed by anti-GST immunoblotting. (C) Schematic design of Cbl-b mutants. (D) Top: IP of proteins from lysates of 293T cells transiently transfected with Flag-tagged Stat6 and HA-tagged Cbl-b, or Cbl-b N1/3 or Cbl-b C2/3 mutants and treated with IL-4 with anti-HA, followed by immunoblot analysis with anti-Flag. Middle and bottom: immunoblot analysis of whole-cell lysates with anti-HA and anti-Flag. (E) Schematic design of Stat6 mutants. (F) Affinity precipitation of proteins from lysates of BALB/c CD4^+^ T cells stimulated with or without anti-CD3 and anti-CD28 with GST-Stat6, GST-Stat6 TAD, or GST-Stat6 SH2 mutant, captured by glutathione sepharose beads, and analyzed by immunoblot analysis with anti-Cbl-b. Immunoblot analysis of whole-cell lysates with anti-Cbl-b was used as a loading control. (G) Top: IP of cytosolic and nuclear extracts of naive WT CD4^+^ T cells stimulated with anti-CD3, anti-CD28, and IL-4 for 0, 5, 15, 30, 60, and 90 min with anti-Stat6, and blotted with anti-Cbl-b and anti-Stat6, respectively. Bottom: Immunoblot analysis of the cytosolic and nuclear extracts with anti-G6DPH (for detection of cytoplasmic protein), and anti-YY1 (for detection of nuclear protein), respectively. Data represent one of three independent experiments.

Having shown that Cbl-b negatively regulates Stat6, we further investigated the mechanism of this regulation by determining whether Cbl-b associates with Stat6. Cbl-b was found to specifically associate with Stat6, but not GATA3, c-Maf, or JunB upon IL-4 and TCR/CD28 stimulation. No association of Stat6 with c-Cbl was observed (Figure 4A). To confirm this, we performed glutathione S-transferase (GST) pull-down assays in which GST-Stat6 (aa 1–680) or GST was incubated with lysates from CD4^+^ T cells that had been stimulated with IL-4, anti-CD3 plus anti-CD28, or both. As shown in Figure 4B, Cbl-b bound to GST-Stat6 only upon TCR/CD28 stimulation, and none of the other E3 ubiquitin ligases tested bound to Stat6. To determine whether Cbl-b also affects the differentiation of other Th subsets, we investigated whether Cbl-b could associate with other Stats. We found that Cbl-b did not bind to Stat1 upon IFN-γ stimulation, to Stat4 upon IL-12 stimulation, or to Stat3 upon IL-6 stimulation (Figure S3A). Therefore, Cbl-b appears to specifically regulate Stat6 during Th2 and Th9 cell differentiation.

To assess whether c-Cbl regulates Th2 cell differentiation, we made use of *c-Cbl*^−/−^ mice (Chiang et al., 2000). Naive CD4^+^ T cells from WT and *c-Cbl*^−/−^ mice were cultured under the Th2 cell differentiation conditions. CD4^+^ T cells lacking c-Cbl showed no Th2-biased phenotype (Figure S3B). Furthermore, although *c-Cbl*^−/−^ CD4^+^ T cells displayed increased phosphorylation of Stat5 at 5 min upon IL-2 stimulation, Stat6 phosphorylation at Y641 was comparable upon IL-4 stimulation in WT and *c-Cbl*^−/−^ T cells (Figure S3C). These data suggest that c-Cbl does not inhibit Th2 cell differentiation. As Stat5 has also been shown to regulate Th2 and Th9 cell differentiation, we assessed Stat5 phosphorylation in response to IL-2 in naive WT and *Cblb*^−/−^ CD4^+^ T cells. Stat5 phosphorylation was not increased in *Cblb*^−/−^ CD4^+^ T cells in response to IL-2 (Figure S3D), suggesting that Cbl-b regulates Th2 and Th9 cell responses independently of Stat5.

It is possible that Cbl-b interacts with Stat6 via two mechanisms: (1) Cbl-b may bind to Stat6 through its TKB domain with phosphotyrosine(s) of Stat6 upon IL-4 stimulation; and (2) phosphotyrosine residues of Cbl-b may bind to the SH2 domain of Stat6 upon TCR/CD28 stimulation. To investigate the modes of interaction, we used 293T cells that lack detectable Stat6 but retain other IL-4R signaling components necessary for Stat6 activation (Mikita et al., 1996). To determine whether IL-4-induced Cbl-b-Stat6 interaction is mediated by Cbl-b’s TKB domain, we transfected 293T cells with hemagglutinin (HA)-tagged Cbl-b or Cbl-b N1/3 (TKB only), or Cbl-b C2/3 (without TKB) mutants (Figure 4C) together with Flag-tagged Stat6, and stimulated them with IL-4. We found that Cbl-b and Cbl-b N1/ 3, but not Cbl-b C2/3, bound to Stat-6, suggesting that IL-4-induced Cbl-b-Stat-6 association requires the Cbl-b TKB domain (Figure 4D). To define whether TCR/CD28-mediated Cbl-b-Stat-6 association is mediated by the interaction of tyrosine-phosphorylated Cbl-b and the Stat6 SH2 domain, we generated GST-Stat6 SH2 recombinant proteins (Figure 4E) and performed a GST pull-down assay. As shown in Figure 4F, GST-Stat6 (aa 1–680) and GST-Stat6 SH2, but not GST-Stat6 TAD, bound to Cbl-b in CD4^+^ T cells stimulated with TCR/ CD28, supporting the notion that the Stat6 SH2 domain interacts with tyrosine residues of Cbl-b. Thus, Cbl-b’s interaction with Stat6 can occur via either its TKB domain or phosphotyrosine residues.

To test whether IL-4-induced Cbl-b-Stat6 interaction interferes with TCR/CD28-induced Cbl-b-Stat6 association, we performed a competition assay using a phosphopeptide derived from IL-4R, which binds to the SH2 domain of Stat6 (Hou et al., 1994; Mikita et al., 1998). The phosphopeptide abrogated both IL-4- and TCR/CD28-induced Cbl-b-Stat6 interaction (Figure S4) because binding of the peptide to the SH2 domain of Stat6 blocks its interaction with the IL-4R. This in turn prevents Stat6 tyrosine phosphorylation, leading to the inability of Stat6 to bind to Cbl-b’s TKB domain and phosphotyrosine residues. In support of this, comparable binding levels of Cbl-b to Stat6 were observed in T cells stimulated with TCR/CD28 or TCR/ CD28/IL-4, suggesting that IL-4-induced Cbl-b-Stat6 association does not interfere with the interaction between Cbl-b and Stat6 induced by TCR/CD28 (Figures 4A and 4B). Furthermore, Cbl-b interacted with Stat6 in the cytosol, but not in the nuclei, as revealed by coimmunoprecipitation (Figure 4G).

### Stat6 Phosphorylation at Y641 Is Required for Its Ubiquitination

The phosphorylation of Stat6 at Y641 is a critical step for its catalytic activity (Mikita et al., 1998). To determine the relationship between Stat6 Y641 phosphorylation and Stat6 ubiquitination, we first examined the kinetics of Stat6 Y641 phosphorylation and ubiquitination, and Cbl-b degradation. Stat6 Y641 phosphorylation and ubiquitination both occurred at 1 min of ligation through CD3/CD28 and IL-4, but Stat6 phosphorylation peaked at 5–15 min and then declined (Figure S5A, top). In contrast, Stat6 ubiquitination peaked at 30–60 min after stimulation. Cbl-b degradation occurred at 15 min and was maintained at lower levels at 60 min of TCR/CD28/IL-4 stimulation (Figure S5A, bottom). These data suggest that Stat6 phosphorylation at Y641 may be required for Stat6 ubiquitination and degradation. This was verified with the Stat6 Y641 mutant (Y641W), which abrogated ubiquitination induced by IL-4 (Figure S5B). Collectively, these data strongly indicate that phosphorylation at Y641 is required for Stat6 ubiquitination.

### Cbl-b Is the E3 Ubiquitin Ligase for Stat6

**Figure 5:**
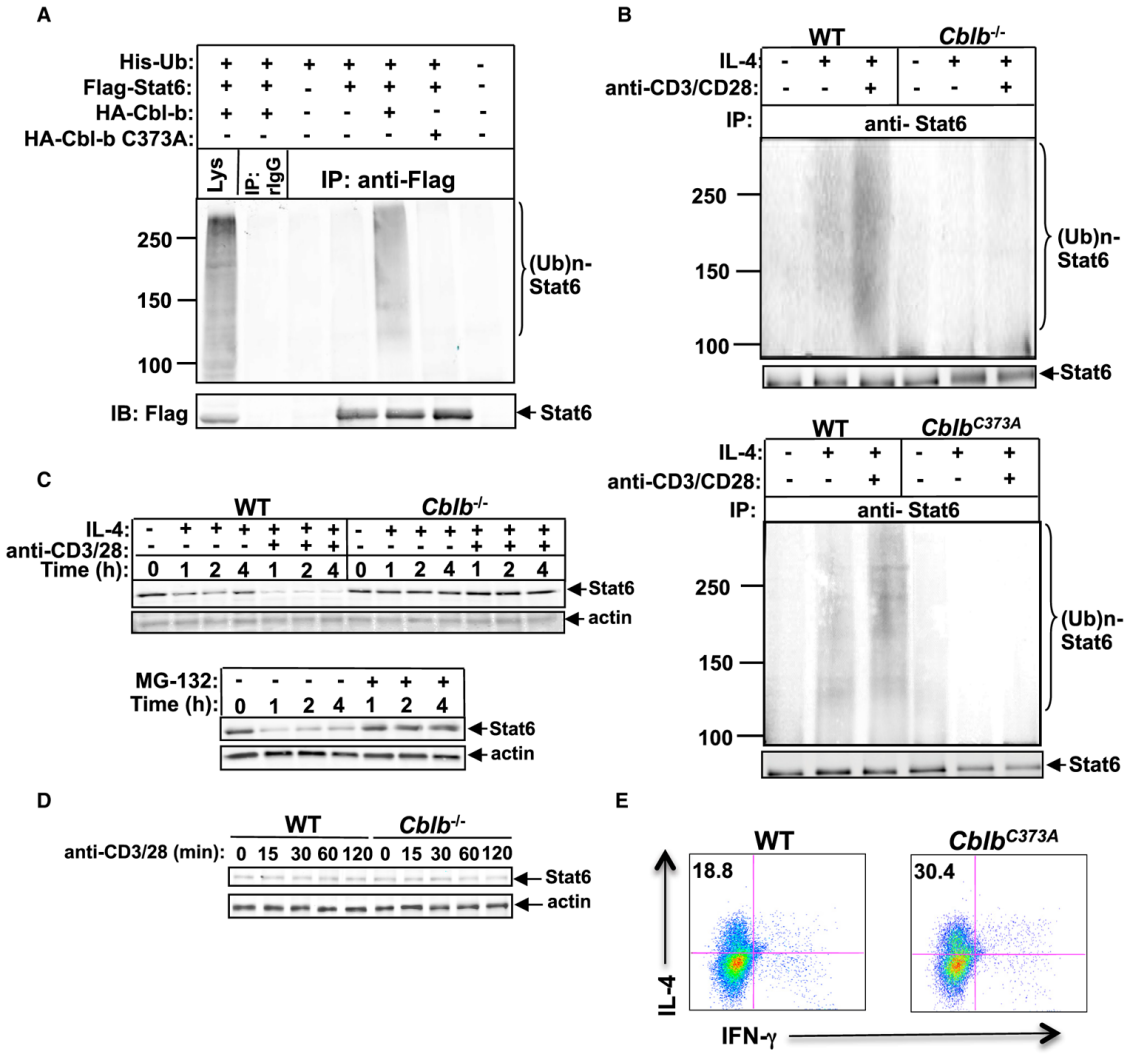
Cbl-b Is the E3 Ubiquitin Ligase for Stat6 (A) IP of proteins from lysates of 293T cells transiently transfected with plasmids encoding with Flag-tagged Stat6, HA-tagged Cbl-b, or Cbl-b C373A mutant, and His-tagged ubiquitin with anti-Flag, followed by immunoblot analysis (IB) with anti-Flag and anti-HA, respectively. (B) IP of proteins from CD4^+^ T cells from WT and *Cblb*^−/−^ mice (top) or WT and *Cblb^C373A^* mice (bottom) pretreated with MG-132 for 30 min, and stimulated with IL-4 in the presence or absence of anti-CD3 and anti-CD28 with anti-Stat6, followed by immunoblotting with anti-ubiquitin and reblotting with anti-Stat6. (C) Top: immunoblot analysis of total protein levels of Stat6 of WT and *Cblb*^−/−^ CD4^+^ T cells stimulated for 1, 2, and 4 hr with IL-4 in the presence or absence of anti-CD3 plus anti-CD28. Bottom: immunoblot analysis of Stat6 protein expression of WT CD4^+^ T cells treated with anti-CD3, anti-CD28, and IL-4 for 1, 2, and 4 hr with or without MG-132. Actin was used as a loading control. (D) Immunoblot analysis of Stat6 protein expression of WT and *Cblb*^−/−^ CD4^+^ T cells stimulated with anti-CD3 and anti-CD28 for 15, 30, 60, and 120 min. (E) Intracellular staining of Th2 cells differentiated in vitro from purified naive CD4^+^CD25^−^CD62L^hi^CD44^lo^ T cells of WT or *Cblb^C373A^* mice. Numbers in the quadrants indicate the percentage of IL-4/IFN-γ-producing cells in the CD4^+^ population. Results are representative of three independent experiments.

To identify whether Cbl-b acts as an E3 ubiquitin ligase for Stat6, we coexpressed HA-tagged Cbl-b or Cbl-b C373A mutant, in which the active-site cysteine at position 373 is substituted with alanine (Ettenberg et al., 2001), with His-tagged ubiquitin and Flag-tagged Stat6 in 293T cells, and stimulated transfected cells with IL-4. Cotransfection with HA-tagged Cbl-b, His-tagged ubiquitin, and Flag-tagged Stat6 resulted in Stat6 ubiquitination, whereas ubiquitination was abrogated with HA-tagged Cbl-b C373A (Figure 5A). To confirm this, CD4^+^ T cells from *Cblb*^−/−^ mice and their WT littermates were pretreated with MG-132 and stimulated with IL-4 in the presence or absence of anti-CD3 and anti-CD28. Cbl-b deficiency impaired IL-4R- or TCR/ CD28/IL-4R-induced Stat6 ubiquitination (Figure 5B, top). In further support of this observation, CD4^+^ T cells isolated from mice expressing the Cbl-b RF C373A mutation (Oksvold et al., 2008) resulted in abrogation of Stat6 ubiquitination (Figure 5B, bottom). Further analysis showed that IL-4-induced Stat6 degradation was exacerbated in TCR/CD28 signaling, and this was completely inhibited by Cbl-b deficiency or the proteasome inhibitor MG-132 (Figure 5C). The key role of IL-4R signaling was further demonstrated by the observation that TCR/CD28 stimulation alone did not induce Stat6 degradation (Figure 5D). To determine whether Cbl-b ubiquitin ligase activity is critical for Th2 development, we performed a Th2 cell differentiation assay using CD4^+^ T cells from the Cbl-b C373A mutant mice. As expected, the Cbl-b C373A mutation resulted in heightened Th2 cell development in vitro (Figure 5E). Collectively, our data establish Cbl-b as a key E3 ubiquitin ligase for regulating Stat6.

### Lysines 108 and 398 Are the Ubiquitination Sites of Stat6

**Figure 6:**
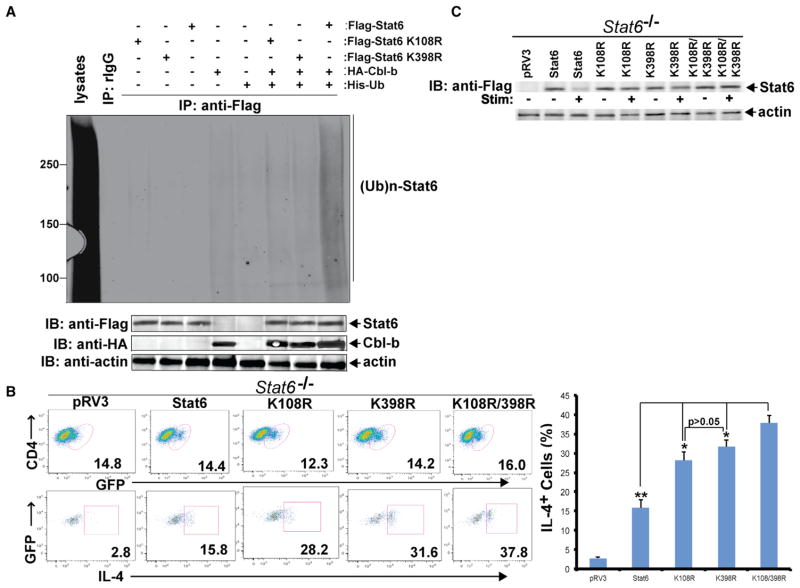
K108 and K398 Are Stat6 Ubiquitination Sites (A) IP of lysates of 293T cells transfected with Flag-tagged Stat6, Stat6 K108R, or Stat6 K398R together with HA-tagged Cbl-b and His-tagged ubiquitin with anti-Flag, and followed by immunoblotting with anti-His. The lysates were blotted with anti-Flag, anti-HA, and anti-actin. (B) Intracellular staining of *Stat6*^−/−^ CD4^+^ T cells reconstituted with Stat6 or Stat6 K108R, K398R, or both, or an empty vector by lentiviral infection, and differentiated under Th2-polarizing condition with anti-iL-4. Numbers in the quadrants indicate the percentage of GFP^+^IL-4-producing cells in the CD4^+^ population. (C) Immunoblotting analysis of lysates of *Stat6*^−/−^ CD4^+^ T cells lentivirally reconstituted with Stat6 or Stat6 K108R, Stat6 K398R, or both, and stimulated with anti-CD3, anti-CD28, and IL-4 for 2 hr. Results are representative of two independent experiments.

To determine the lysine residue(s) responsible for Stat6 ubiquitination, we first used the Bayesian Discriminant Method (BDM-PUB; http://bdmpub.biocuckoo.org/) to predict potential ubiquitination sites. We identified 22 potential lysine residues within Stat6, and chose the 14 residues with scores higher than 1.0 (K108, K194, K199, K252, K307, K361, K367, K369, K374, K374, K398, K618, K621, and K647; Figure S6A). To determine the Stat6 ubiquitination site(s), we made point mutations (K to R) at each lysine. Only mutations at K108 and K398 significantly diminished Stat6 ubiquitination in 293T cells induced by IL-4 stimulation (Figures 6A and S6B), indicating that K108 and K398 are the ubiquitination sites. To define the biological relevance of K108 and K398, we generated Stat6 mutants carrying K108R, K398R, or both, and reconstituted *Stat6*^−/−^ CD4^+^ T cells with WT Stat6 or Stat6 K108R, K398R, or both. We found that more *Stat6*^−/−^ CD4^+^ T cells reconstituted with Stat6 K108R, K398R, or K108R/K398R differentiated into Th2 cells compared with those reconstituted with WT Stat6 (Figure 6B). Consistent with this observation, stimulation of *Stat6*^−/−^ CD4^+^ T cells reconstituted with WT Stat6 with TCR/CD28/IL-4 induced Stat6 degradation, but Stat6 degradation was significantly reduced or abrogated in *Stat6*^−/−^ CD4^+^ T cells reconstituted with Stat6 K108R, K398R, or K108R/K398R (Figure 6C). These findings clearly identify the Stat6 ubiquitination sites and verify the importance of Stat6 ubiquitination in the regulation of Th2 cell differentiation.

### Introducing Stat6 Deficiency Abrogates Hyper-Th2 Responses but Only Partially Attenuates Th9 Responses in Cblb^−/−^ Mice

**Figure 7:**
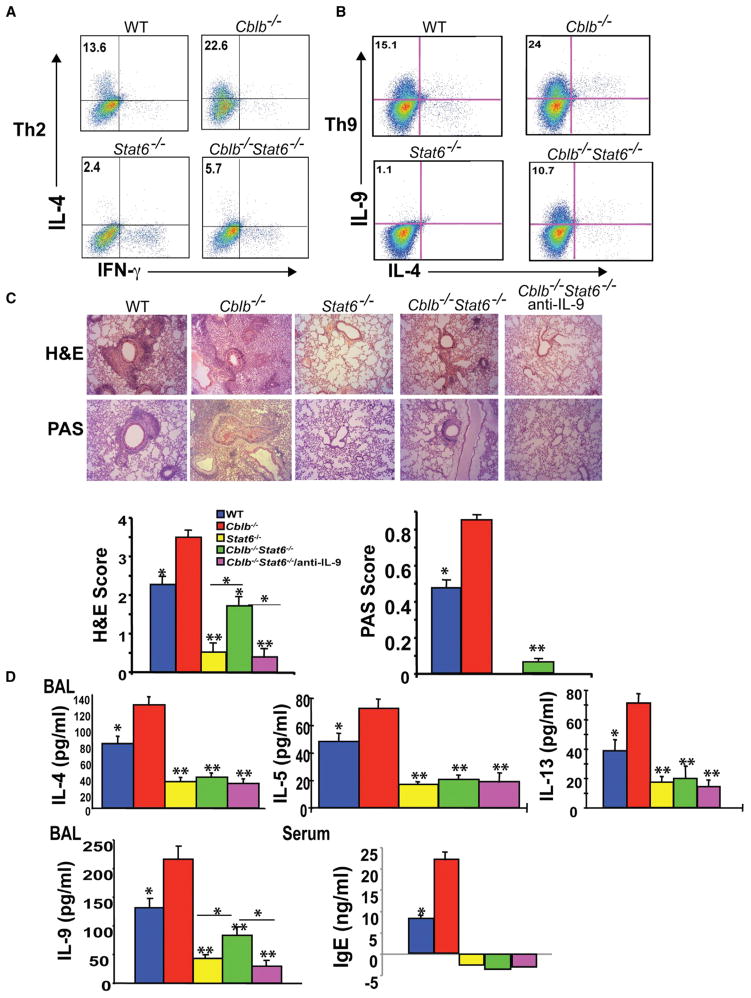
Loss of Stat6 Abrogates Hyper-Th2 and Th9 Responses in *Cblb*^−/−^ Mice (A and B) Intracellular staining of Th2 and Th9 cells differentiated in vitro from purified naive CD4^+^CD25^−^CD62L^hi^CD44^lo^ T cells of WT, *Cblb*^−/−^, *Stat6*^−/−^, and *Cblb*^−/−^*Stat6*^−/−^ mice. Numbers in the quadrants indicate the percentage of IL-4/IFN-γ-producing cells (A) or IL-9/IL-4-producing cells (B) in the CD4^+^ population. (C) H&E and PAS staining of the lungs of WT, *Cblb*^−/−^, *Stat6*^−/−^, and *Cblb*^−/−^*Stat6*^−/−^ mice treated or untreated with anti-iL-9. Original magnification ×100 (*p < 0.05 and **p < 0.01, compared with *Cblb*^−/−^ mice; Mann-Whitney test). (D) ELISA of IFN-γ, IL-4, IL-5, IL-9, and IL-13 concentrations in the BAL fluid and IgE in the serum of OVA-sensitized WT, *Cblb*^−/−^, *Stat6*^−/−^, and *Cblb*^−/−^*Stat6*^−/^− mice treated or untreated with anti-iL-9. Data represent three independent experiments (mean ± SD).

To further elucidate the role of Stat6 in hyper-Th2 and Th9 responses in *Cblb*^−/−^ mice, we introduced Stat6 deficiency into *Cblb*^−/−^ mice. If the heightened Th2 and Th9 responses caused by Cbl-b deficiency are mediated by Stat6, one would predict that loss of Stat6 should abrogate these hyperresponses in *Cblb*^−/−^ mice. As predicted, the heightened Th2 cell differentiation in vitro in the absence of Cbl-b was abrogated by introducing Stat6 deficiency (Figure 7A). Unexpectedly, loss of Stat6 only moderately affected Th9 cell differentiation by *Cblb*^−/−^ CD4^+^ T cells (Figure 7B). To further confirm whether Stat6 deficiency attenuates aberrant airway inflammation and Th2 and Th9 responses in vivo, we immunized WT, *Cblb*^−/−^, *Stat6*^−/−^, and *Cblb*^−/−^*Stat6*^−/−^ mice with OVA in alum. Stat6 deficiency greatly diminished heightened airway inflammation and PAS^+^ airway cell accumulation (Figure 7C), and inhibited total serum IgE titers, eosinophil infiltration, and Th2 cytokines (IL-4, IL-5, and IL-13) in the BAL of *Cblb*^−/−^ mice (Figure 7D). In contrast, Stat6 deficiency only moderately attenuated IL-9 levels in the BAL of *Cblb*^−/−^ mice (Figure 7D). These data are consistent with the fact that airway inflammation was completely diminished in *Stat6*^−/−^ mice, whereas some residual airway inflammation was observed in *Cblb*^−/−^ *Stat6*^−/−^ mice (Figure 7C). To further confirm whether the residual airway inflammation in *Cblb*^−/−^ *Stat6*^−/−^ mice was due to Th9 responses, we treated *Cblb*^−/−^ *Stat6*^−/−^ mice with a neutralizing anti-iL-9 antibody. As expected, anti-iL-9 treatment completely abrogated residual airway inflammation in *Cblb*^−/−^ *Stat6*^−/−^ mice (Figure 7C).

Stat6 has been shown to be required in airway epithelial and smooth muscle cells in addition to Th2 and Th9 cells (Matsukura et al., 2001; Perkins et al., 2011). To further verify that Stat6 is the downstream target of Cbl-b in T cells, we performed adoptive transfer of naive CD4^+^ T cells from WT, *Cblb*^−/−^, *Stat6*^−/−^, and *Cblb*^−/−^ *Stat6*^−/−^ mice into BALB/c nude mice, followed by immunization with OVA in alum and challenge with an aerosol form of OVA. Consistent with the data shown in Figures 2E and 2F, BALB/c nude mice that received *Cblb*^−/−^ CD4^+^ T cells developed severe airway inflammation upon immunization and airway challenge with OVA, and this heightened airway inflammation was significantly reduced, but not abrogated, when Stat6 deficiency was introduced (Figure S7). Taken together, our data demonstrate that Stat6 is the target for Cbl-b during Th2 cell differentiation both in vitro and in vivo. Our data also suggest that Th9 cell differentiation regulated by Cbl-b is mediated by both Stat6-dependent and -independent mechanisms.

## DISCUSSION

*Cblb*^−/−^ mice are highly susceptible to autoimmunity (Chiang et al., 2000; Jeon et al., 2004), which is believed to be mediated by Th17 (Bettelli et al., 2007; Korn et al., 2009). Therefore, it is possible that Cbl-b may also regulate Th17 cell differentiation. However, our in vitro studies suggest that loss of Cbl-b does not affect Th17 cell differentiation, which contradicts a recent report in which IL-17 production, but not IL-17 intracellular staining, was used as a readout for Th17 cells (Gruber et al., 2009). It is possible that Cbl-b deficiency in other cell types, such as B cells and monocytes/macrophages, may affect in vivo Th17 responses. Indeed, Cbl-b deficiency results in hyperactivation of B cells via BCR (Sohn et al., 2003) or CD40 (Qiao et al., 2007), and monocytes via TLR-4 (Bachmaier et al., 2007). The heightened production of proinflammatory cytokines via B cells and/or monocytes/macrophages in the absence of Cbl-b may eventually affect Th17 responses in vivo. In keeping with this scenario, we have found that *Cblb*^−/−^ mice are highly susceptible to experimental autoimmune myocarditis (EAM), but it seems that the heightened Th17 responses in EAM observed in *Cblb*^−/−^ mice are not due to T cell-intrinsic loss of Cbl-b (our unpublished data). Nevertheless, the reconstitution of BALB/c nude mice with *Cblb*^−/−^ CD4^+^ T cells leads to severe airway inflammation upon OVA immunization and challenge associated with heightened Th2 and Th9 responses (Figure 2F), indicating a T cell-intrinsic role for Cbl-b in the regulation of Th2 and Th9 cell differentiation. In support of this, in vitro Th differentiation assays using naive CD4^+^ T cells showed an aberrant differentiation of *Cblb*^−/−^ T cells into the Th2 and Th9 cell lineage (Figures 1B–1D). Therefore, our data collectively indicate that Cbl-b specifically inhibits Th2 and Th9 cell differentiation, providing a potential pharmaceutical target for allergic asthma. Our data differ from that reported by Oh et al. (2011), who failed to observe heightened airway inflammation and Th2 and Th9 responses in *Cblb*^−/−^ mice upon OVA/alum immunization protocol. The increased airway inflammation they observed in *Cblb*^−/−^ mice upon intranasal challenge was predominantly mediated by a Th1 response in the lung. This discrepancy may be due to the different genetic backgrounds of the mice (B6 versus BALB/c) and/or the doses of OVA used.

Recent studies indicate that Stat6 appears to be required for Th9 cell differentiation (Goswami et al., 2012; Kaplan, 2013). Surprisingly, although we observed a significant increase in Th9 cell responses in vitro and in vivo in the absence of Cbl-b (Figures 1 and 2), Stat6 deficiency only partially reduced this Th9 response in vitro and in vivo (Figures 7 and S7). This is supported by evidence that some residual airway inflammation was still present in *Cblb*^−/−^ *Stat6*^−/−^ mice (Figures 7 and S7). Therefore, Cbl-b, through targeting Stat6 for ubiquitination, inhibits Th2 responses, but Cbl-b inhibits Th9 responses via both Stat6-dependent and -independent mechanisms.

We found that Stat6 specifically binds Cbl-b upon IL-4 stimulation, and that TCR/CD28 stimulation strengthens this interaction. It seems that Cbl-b interacts with Stat6 via two different mechanisms depending upon the stimuli. IL-4-induced Cbl-b-Stat6 association requires the Cbl-b TKB domain, which potentially interacts with tyrosine residues of Stat6, whereas TCR/CD28-induced Cbl-b-Stat6 interaction may be mediated by phosphorylated Cbl-b C-terminal tyrosine residues and the SH2 domain of Stat6. In keeping with these observations, we found that Stat6 undergoes proteasomal modification upon IL-4 treatment, which is heightened by TCR/CD28 stimulation, whereas this process is abrogated in the absence of Cbl-b (Figure 5C). Although it is unknown why TCR/CD28 stimulation does not induce Stat6 ubiquitination but induces a strong interaction between Cbl-b and Stat6, it is possible that the orientation of this interaction is unable to promote the transfer of ubiquitin, whereas the binding of the Cbl-b TKB domain to phosphotyrosine residues of Stat6 upon IL-4 stimulation provides a correctly aligned interaction that can allow Stat6 ubiquitination.

Our data demonstrate that Stat6 is ubiquitinated at K108 and K398 by Cbl-b, and that Stat6 ubiquitination is a critical post-translational regulatory mechanism for Stat6. Although it was previously reported that Stat6 may be modified by calcium-dependent proteases via a proteolytic mechanism (Zamorano et al., 2005), it remained unknown whether Stat6 is regulated via the ubiquitin-proteasome pathway during Th2 and Th9 cell differentiation. Our study demonstrates that Stat6 is regulated by the ubiquitination-proteasome pathway, and to identify Cbl-b as the E3 ubiquitin ligase that targets Stat6. Therefore, we have identified a mechanism for Cbl-b in regulating Th2 and Th9 cell differentiation that may serve as an important therapeutic target for allergic diseases, including asthma.

## EXPERIMENTAL PROCEDURES

### Mice

Details regarding the mice used in this study are provided in the Supplemental Experimental Procedures. All experimental protocols followed NIH guidelines and were approved by the institutional animal care and use committees of the University of Chicago and Ohio State University. All of the mice were 6–10 weeks old when they were used for experiments.

### In Vitro Th1, Th2, Th9, and Th17 Differentiation Assays

Naive CD4^+^ T cells isolated from WT and *Cblb*^−/−^ mice were stimulated with plate-bound anti-CD3 (2 μg/ml) and anti-CD28 (1 μg/ml) in the presence of Th1, Th2, Th17, or Th9 cytokine cocktails as previously described (Ying et al., 2010; Chang et al., 2010). Details are provided in the Supplemental Experimental Procedures.

### Immunoprecipitation and Western Blotting

The conditions for immunoprecipitation and immunoblotting were described previously (Zhang et al., 2002; Li et al., 2004; Qiao et al., 2008). Details are provided in the Supplemental Experimental Procedures.

### Asthma Induction

Mice (five mice/group) were immunized by intraperitoneal (i.p.) injection of OVA (100 μg/ml; Sigma-Aldrich) adsorbed to 2 mg of an aqueous solution of aluminum hydroxide and magnesium hydroxide (Alum; Fischer Scientific) on day 0 and day 14. After 21 days, challenge doses of OVA were given through the airways. Details are provided in the Supplemental Experimental Procedures.

### Statistical Analysis

A two-tailed Student’s t test was applied for statistical comparison of two groups or, when appropriate, two-way ANOVA followed by Bonferroni’s post hoc test for multiple comparisons and a Mann-Whitney test for nonparametric data (asthma scoring). A p value of 0.05 or less was considered significant.

## Supplementary Material

01

02
