# Supplementary material for: E3 Ubiquitin Ligase Cbl-b Suppresses Proallergic T Cell Development and Allergic Airway Inflammation
Source: Cell Rep. Author manuscript; Available in PMC 2015 Feb 27. (PMC3969736; doi:10.1016/j.celrep.2014.01.012)
Supplement: 01 [file NIHMS558467-supplement-01.pdf]

SUPPLEMENTAL INFORMATION

The supplemental data includes 7 supplemental figures with legends, supplemental experimental procedures, and supplemental references.

A

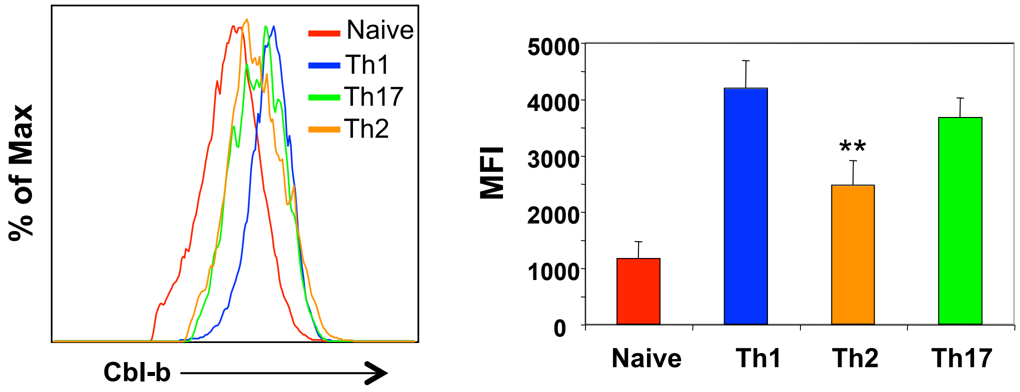
Naive
Th1
Th17
Th2
% of Max
Cbl-b
5000
4000
3000
2000
1000
0
MFI
**
Naive
Th1
Th2
Th17

B

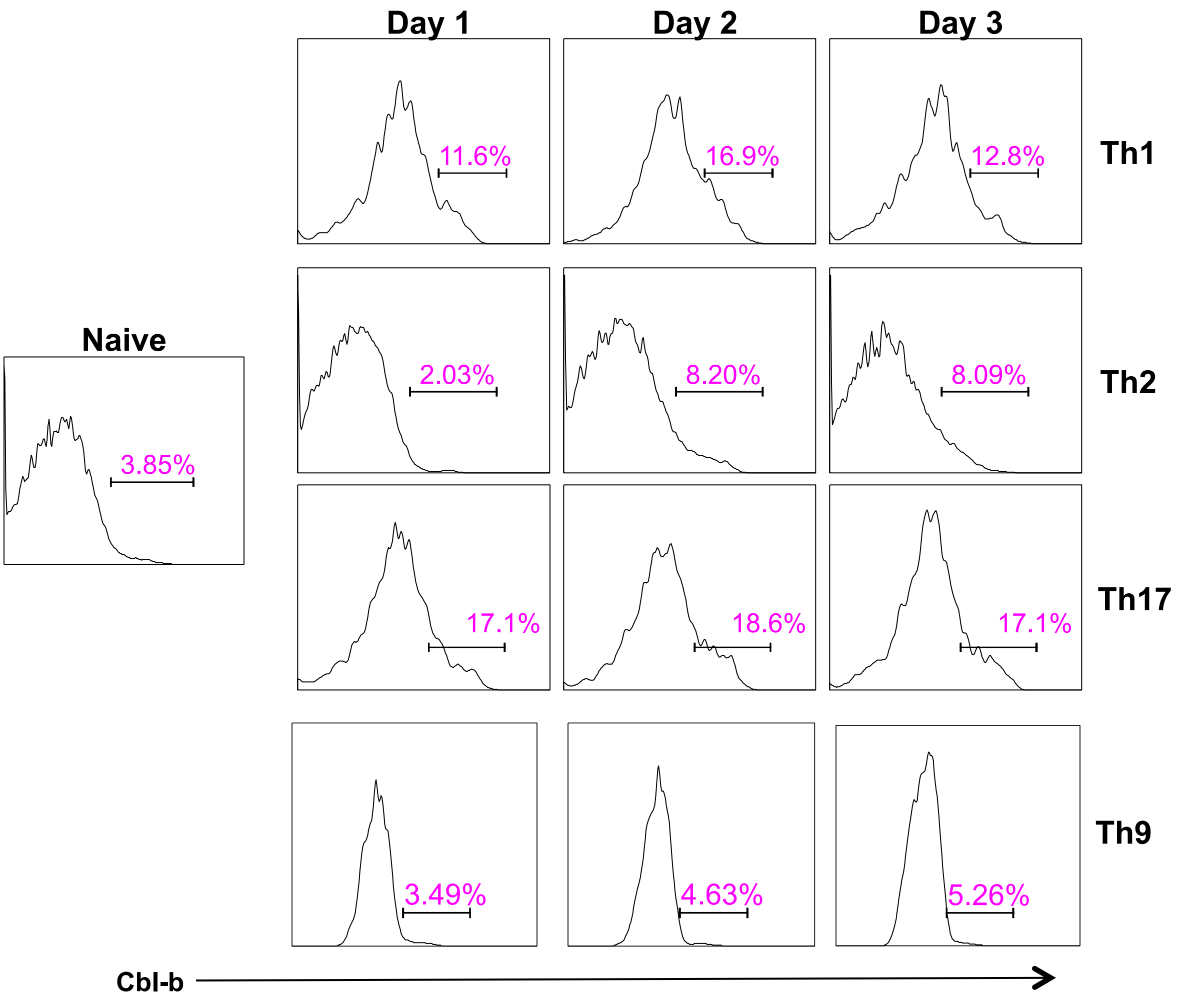
Day 1
Day 2
Day 3
Th1
11.6%
16.9%
12.8%
Naive
3.85%
Th2
2.03%
8.20%
8.09%
Th17
17.1%
18.6%
17.1%
Th9
3.49%
4.63%
5.26%
Cbl-b

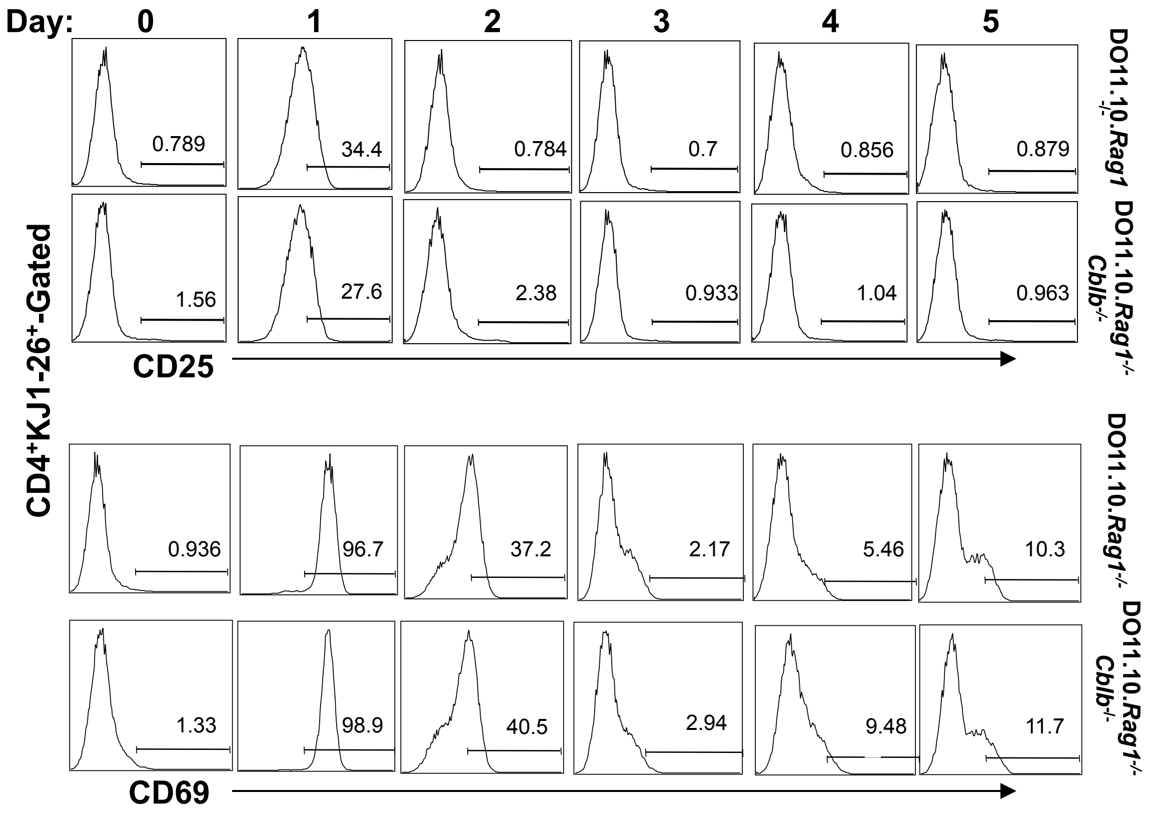

Day: 0 1 2 3 4 5
CD4+KJ1-26+-Gated
DO11.10.Rag1-/-
DO11.10.Rag1-/- Cblb-/-
0.789 34.4 0.784 0.7 0.856 0.879
1.56 27.6 2.38 0.933 1.04 0.963
CD25
0.936 96.7 37.2 2.17 5.46 10.3
1.33 98.9 40.5 2.94 9.48 11.7
CD69

Qiao et al., Figure S3

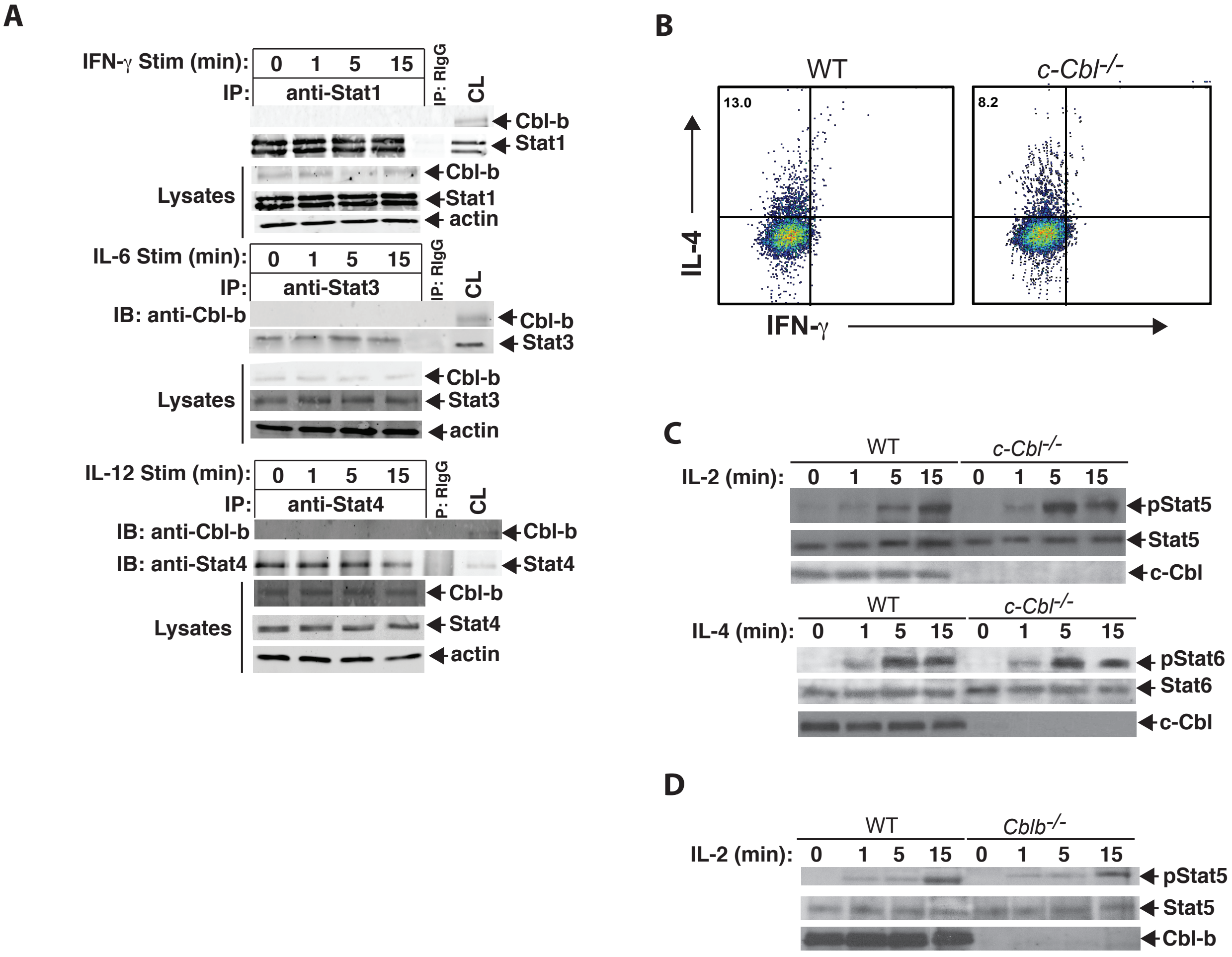

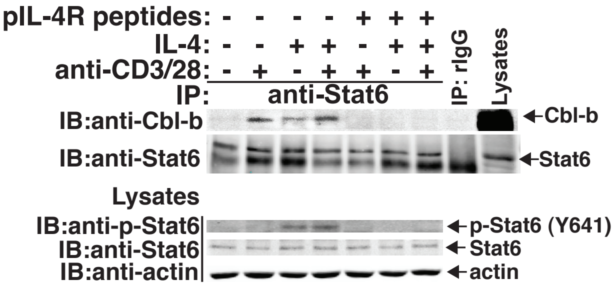
pIL-4R peptides: - - - - + + +
IL-4: - - + + - + +
anti-CD3/28: - + - + + - +
IP: anti-Stat6
IP: rIgG
Lysates
IB:anti-Cbl-b
Cbl-b
IB:anti-Stat6
Stat6
Lysates
IB:anti-p-Stat6
p-Stat6 (Y641)
IB:anti-Stat6
Stat6
IB:anti-actin
actin

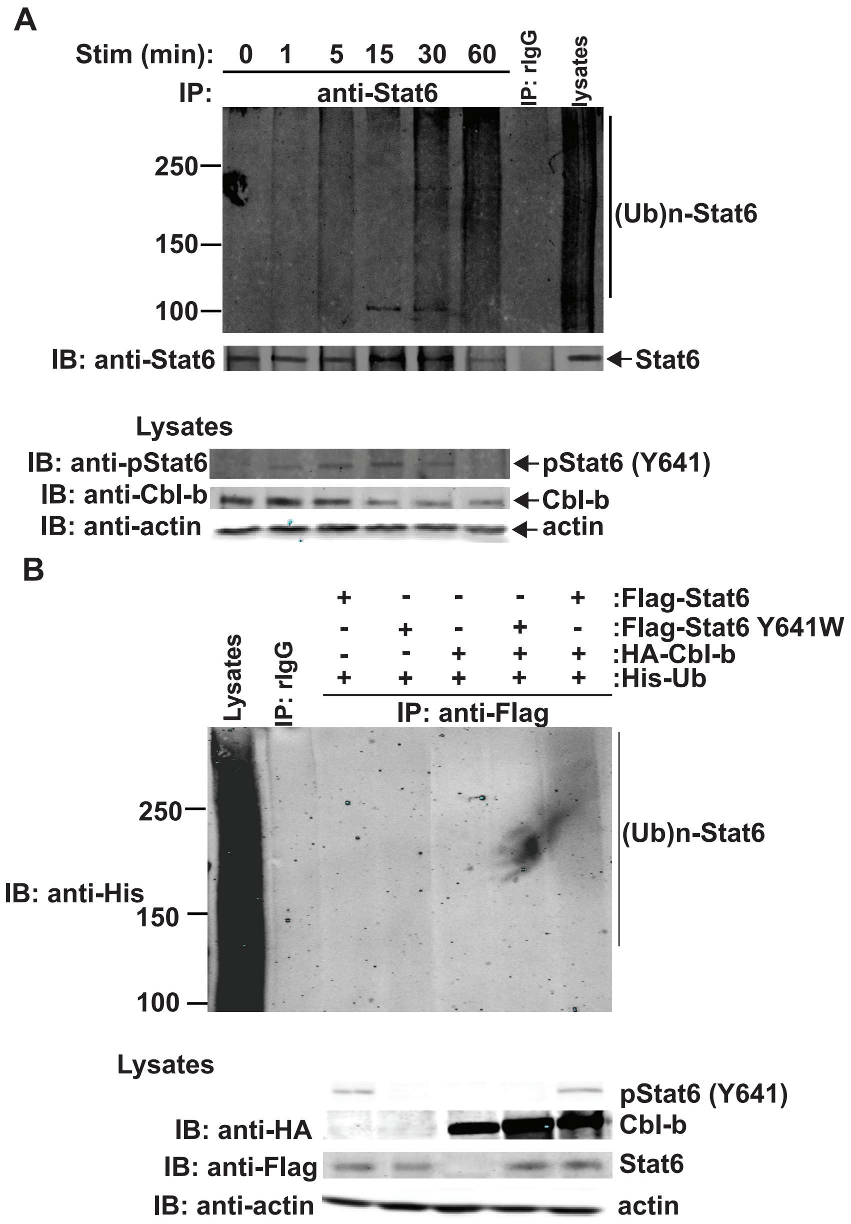
A
Stim (min): 0 1 5 15 30 60
IP: anti-Stat6
IP: rIgG
lysates
250
150
100
(Ub)n-Stat6
IB: anti-Stat6
Stat6
Lysates
IB: anti-pStat6
pStat6 (Y641)
IB: anti-Cbl-b
Cbl-b
IB: anti-actin
actin
B
:Flag-Stat6
:Flag-Stat6 Y641W
:HA-Cbl-b
:His-Ub
Lysates
IP: rIgG
IP: anti-Flag
250
150
100
IB: anti-His
(Ub)n-Stat6
Lysates
pStat6 (Y641)
IB: anti-HA
Cbl-b
IB: anti-Flag
Stat6
IB: anti-actin
actin

A

Peptide	Position	Score
QILQGEKKAVMEQFR	108	1.15
TGELEAAKALVLKRI	194	2.48
AAKALVLKRIQIWKR	199	1.08
AGGELEPKTRASLTG	252	1.70
RFLGAPAKPPLVRAD	307	1.93
NCCSALFKNLLLKKI	361	2.63
FKNLLLKKIKRCERK	367	1.03
NLLLKKIKRCERKGT	369	1.54
KIKRCERKGTESVTE	374	1.38
SFTLGPGKLPIQLQA	398	1.49
QLKNLYPKKPKDEAF	618	1.52
NLYPKKPKDEAFRSH	621	1.85
GYVPATIKMTVERDQ	647	1.62

B

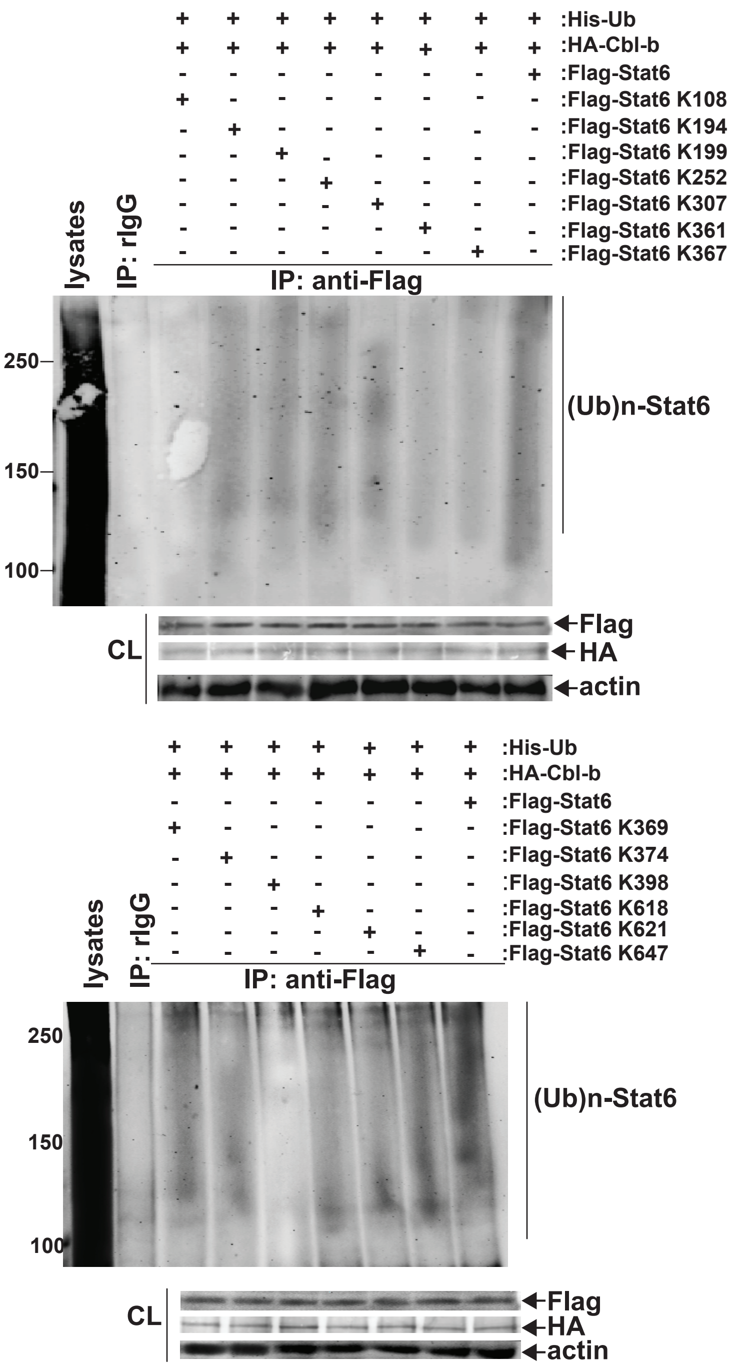

Qiao, et al., Figure S7

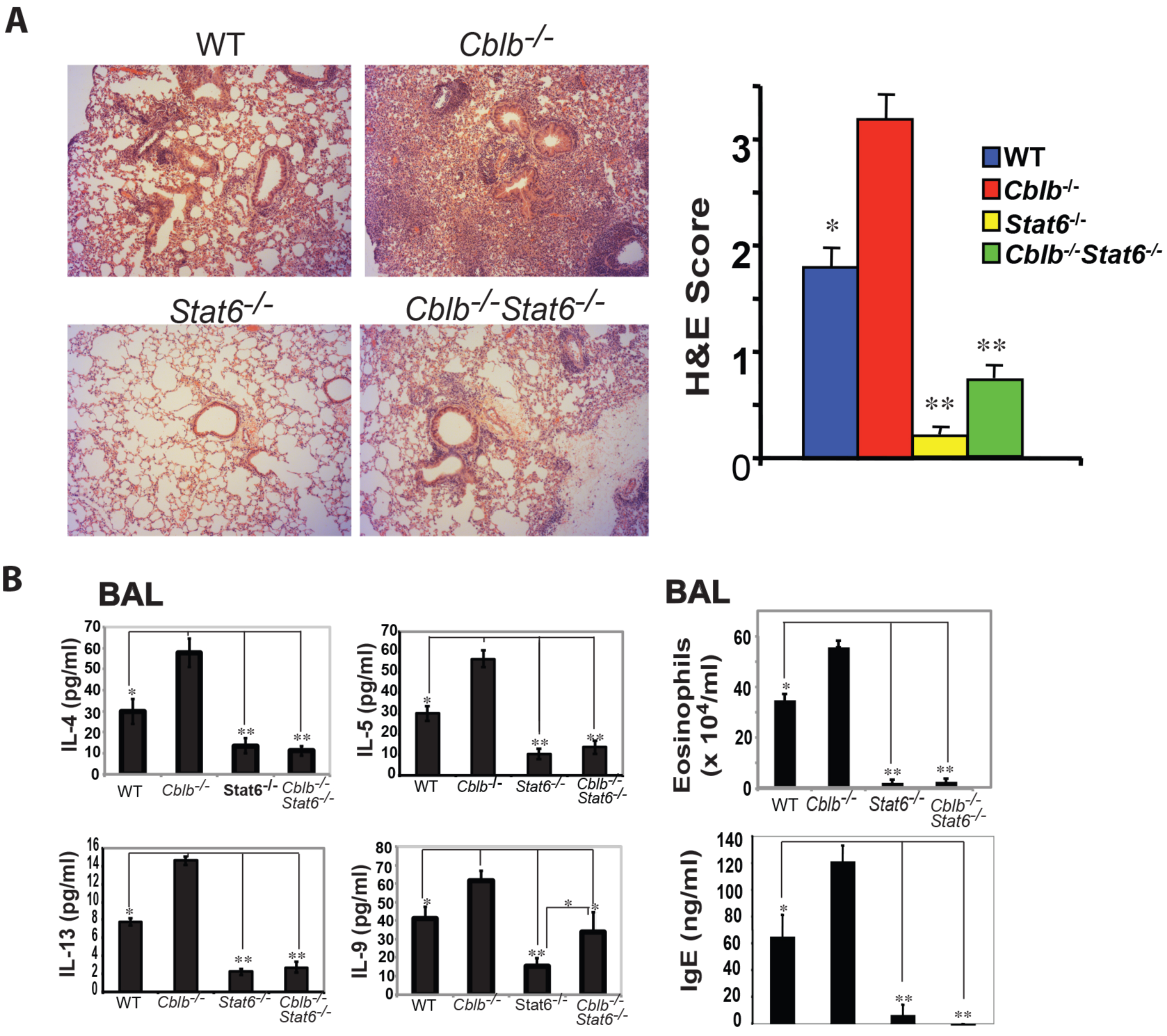

SUPPLEMENTAL FIGURE LEGENDS:

Figure S1. Cbl-b expression is highly expressed in Th1 and Th17 but not Th2 cells, Related to Figure 1.

(A) Naïve $CD4^{+}CD25^{-}$ T cells were cultured under Th1-, Th2-, or Th17-polarized conditions. By the end of differentiation, the cells were collected, surface-stained with anti-CD4, intracellularly stained with anti-IFN-γ, anti-IL-4, and anti-IL-17, respectively, and then intracellularly stained with anti-Cbl-b followed by fluorescence-conjugated rabbit-anti-mouse IgG1. The expression of Cbl-b in Th1 ($CD4^{+}IFN\text{-}\gamma^{+}$), Th2 ($CD4^{+}IL\text{-}4^{+}$), and Th17 ($CD4^{+}IL\text{-}17^{+}$) cells was determined by flow cytometry. Naïve $CD4^{+}$ T cells were used as a control. (*$p<0.01$ compared to Th1 or Th17; Student *t* test).

(B) Naïve $CD4^{+}CD25^{-}$ T cells were cultured under Th1-, Th2-, Th9-, or Th17-polarized conditions, and the cells were collected on day 1, 2, and 3, and Cbl-b expression in differentiating Th1, Th2, Th9, and Th17 cells was determined as described above.

Figure S2. Immunization of OVA in alum leads to comparable antigen-specific T cell activation in WT mice receiving naïve $CD4^{+}$ T cells of DO11.10 mice in the presence or absence of Cbl-b, Related to Figure 2.

WT BALB/c mice receiving naïve $CD4^{+}$ T cells from DO11.10.*Rag1*$^{-/-}$ or DO11.10.*Rag1*$^{-/-}$.*Cblb*$^{-/-}$ mice were immunized with 100 μg/ml in alum. Three mice

were sacrificed each day for five days. The expression of activation markers CD25 and CD69 in CD4$^+$ T cells was determined by flow cytometry.

Figure S3. Cbl-b but not c-Cbl regulates Th2 cell differentiation, Related to Figure 4.

(A) BALB/c CD4$^+$ T cells were stimulated with IFN-γ, IL-6, or IL12 for 1, 5, and 15 min, and lysed in 0.5% NP-40 lysis buffer. The cell lysates were immunoprecipitated with anti-Stat1, anti-Stat3, and anti-Stat4, respectively, and blotted with anti-Cbl-b. Cell lysates were used as a positive control, and normal rabbit IgG was used as a negative control for immunoprecipitation. The membranes were reprobed with anti-Stat1, anti-Stat3, and anti-Stat4, respectively. The cell lysates from each sample were blotted with Abs against respective Stats, Cbl-b, and actin.

(B) Naïve CD4$^+$ T cells from WT and *c-Cbl*$^{-/-}$ mice are cultured under Th2-polarizing condition, and Th2 cells were determined.

(C) Naïve CD4$^+$ T cells from WT and *c-Cbl*$^{-/-}$ mice were stimulated with IL-2 or IL-4 for 1, 5, and 15 min, and lysed. The cell lysates were blotted using phospho-antibodies against Stat5 (Y694) and Stat6 (Y641), respectively.

(D) Naïve CD4$^+$ T cells from WT and *Cblb*$^{-/-}$ mice were stimulated with IL-2 for 1, 5, and 15 min, and lysed. Phospho-Stat5 was determined by immunoblotting.

Figure S4. Phospho-peptide derived from IL-4Rα abolishes the binding of Stat6 to Cbl-b, Related to Figure 4.

BALB/c $CD4^+$ T cells were pre-incubated with a phospho-peptide derived from IL-4Rα (ASSGEEGPYKPFQDLI) for 30 min, stimulated with TCR/CD28/IL-4, and lysed. The cell lysates were immunoprecipitated with anti-Stat6, and blotted with anti-Cbl-b and anti-Stat6. The cell lysates from each sample were blotted with anti-phospho-Stat6 (Y641), and anti-actin, respectively.

Figure S5. Stat6 Y641 is required for its ubiquitination, Related to Figure 5.

(A) WT $CD4^+$ T cells were stimulated with anti-CD3, anti-CD28, and IL-4 for 1, 5, 15, 30 and 60 min. The cell lysates were immunoprecipitated with anti-Stat6, and blotted with anti-ubiquitin, anti-phospho-Stat6 (Y641), and anti-Stat6, respectively. An aliquot of the samples was blotted with anti-Cbl-b and anti-actin.

(B) 293T cells were transfected with WT Stat6 or Stat6 Y641W mutant, together with HA-tagged Cbl-b, and His-tagged ubiquitin. The cells were then stimulated with IL-4, and lysed in RIPA buffer. The cell lysates were immunoprecipitated with anti-Flag, and blotted with anti-His. The cell lysates were also blotted with anti-HA, anti-Flag, and anti-actin, respectively.

Figure S6. Stat6 is ubiquitinated at K108 and K398, Related to Figure 6.

(A) Prediction of Stat6 ubiquitination sites by computational software**.**

(B) 293T cells were transfected with Flag-tagged WT Stat6, or Stat6 lysine mutants described above, together with HA-tagged Cbl-b and His-tagged ubiquitin. The transfected cells were stimulated with IL-4, and lysed in RIPA buffer. The cell lysates were immunoprecipitated with anti-Flag, and blotted with

anti-His. The cell lysates were blotted with anti-Flag, anti-HA, and anti-actin, respectively.

Figure S7. T cell-intrinsic loss of Stat6 abrogates hyper-Th2 but only partially attenuates Th9 responses in the absence of Cbl-b, Related to Figure 7.

(A) BALB/c nude (n=4) were adoptively transferred (i.v.) with naïve $CD4^+$ T cells (5 $x10^6$) from WT, *Cblb*$^{-/-}$, *Stat6*$^{-/-}$, and *Cblb*$^{-/-}$*Stat6*$^{-/-}$ mice, permitted to equilibrate 30 days to avoid homeostatic proliferation, and immunized and challenged with OVA. Airway inflammation determined by H&E staining.

(B) The serum IgE and BAL eosinophils and cytokines IL-4, IL-5, IL-9, and IL-13 were determined by ELISA.

SUPPLEMENTAL EXPERIMENTAL PROCEDURES

Mice

WT BALB/c, *Stat6*$^{-/-}$, and DO11.10 mice were purchased from The Jackson Laboratory (Bar Harbor, ME). *Cblb*$^{-/-}$ mice were described previously (Bachmaier et al., 2000), and have been backcrossed onto the BALB/c background for 14 generations. BALB/c *Cblb*$^{-/-}$ mice were crossed onto DO11.10.*Rag1*$^{-/-}$ or *Stat6*$^{-/-}$ to generate DO11.10.*Rag1*$^{-/-}$*Cblb*$^{-/-}$ mice or *Cblb*$^{-/-}$*Stat6*$^{-/-}$ mice. C*blb*^{C373A} knockin mice were generated by a targeting vector introducing the Cys (TGC) to Ala (GCG) substitution at amino acid 373 using PCR fragments generated from 129Sv/J genomic DNA (Oksvold et al., 2008). *c-Cbl*$^{-/-}$ mice were described previously (Chiang et al., 2000).

Reagents

Purified anti-mouse CD3 (145-2C11) and anti-mouse CD28 (37.51) mAbs and all the antibodies used in flow cytometry including anti-phospho-Stat6 (Tyr641) (J71-773.58.11) were purchased from BD PharMingen (San Diego, CA). Protein G-Sepharose was purchased from GE Healthcare (Piscataway, NJ). Anti-Itch (H-110), anti-Nedd4 (D-17), anti-TRAF-2 (C-20), anti-TRAF-6 (H-257), anti-Stat-6 (M-20), anti-Cbl-b (G-1), anti-HA (D-8 and Y-11), anti-His (H-3), anti-GATA3 (HG3-31), anti-c-Maf (M-153), anti-T-bet (4B10), anti-JunB (N-17), anti-p-JAK1 (Tyr1022), anti-p-JAK3 (Tyr980), anti-c-Cbl (C-15), anti-Stat1 (M-22), anti-Stat3 (C-20), anti-Stat4 (C-20), anti-IRF4 (M17), and anti-ubiquitin (P4D1) were purchased from Santa Cruz Biotechnology (Santa Cruz, CA). HRP-conjugated

goat anti-rabbit IgG or rabbit anti-mouse IgG were purchased from Kirkegaard & Perry Laboratories (Gaithersburg, MD). A phosphpo-peptide (ASSGEEGPYKPFQDLI) derived from IL-4Rα was synthesized by Peptide 2.0 (Chantilly, VA).

Plasmids and transfection

Cbl-b cDNAs encoding full-length (FL) or different mutant Cbl-b with an HA epitope in pCEFL were described previously (Ettenberg et al., 2001). His6-tagged ubiquitin plasmid was a gift from Dr. Dirk Bohmann (University of Rochester, Rochester, NY). Stat6 cDNA encoding full-length or different mutant Stat6 with a Flag tag were obtained from Dr. Mark H. Kaplan (Indiana University, Indianapolis, IN). Large scale of plasmids were prepared using an endofree large scale plasmid extraction kit (Qiagen, Valencia, CA), and transfected into 293T cells by calcium precipitation. For expression and purification of GST-fusion proteins, plasmids were transformed into T7 Express Competent *Escherichia coli* cells (New England Biolabs Inc., Ipswich, MA), induced at OD_{600} = 0.6-0.7 and then grown at 37°C for 4 h. Soluble proteins were purified with fast flow glutathione sepharose beads (GE Lifesciences).

Site-directed mutagenesis of Stat6 lysine mutants

Site-directed mutagenesis of Stat6 K to R mutants was performed at Mutagenex Laboratories (Hillsborough, NJ) as described (Ko and Ma, 2005).

Retrovirus- and lentivirus-mediated gene transfer

pGFP-RV containing IRES-regulated GFP, and pGFP-RV-GATA3 were gifts from K. Murphy (Washington University, St. Louis, MO) (Ouyang et al., 1998). Naïve $CD4^{+}CD25^{-}CD62L^{hi}CD44^{lo}$ T cells from WT and *Cblb*$^{-/-}$ mice were FACS sorted and activated with anti-CD3 and anti-CD28 in the presence 10 μg/ml anti-IL-4. Twenty-four hours after activation, the cells were infected with retroviruses expressing GATA3-GFP or control empty vector (containing only *IRES-GFP*). Three days after infection, the cells were restimulated with PMA and ionomycin in the presence of Golgi-stop for 5 hr, after which IL-4-producing cells were analyzed with intracellular staining on a GFP^{+} gate.

The pRV3-GFP-Stat6, pRV3-GFP-Stat6 K108R, pRV3-GFP-Stat6 K398R, and pRV3-GFP-Stat6 K108/398R constructs were generated by Mutagenex Laboratories. Naïve $CD4^{+}CD25^{-}CD62L^{hi}CD44^{lo}$ T cells from *Stat6*$^{-/-}$ mice were activated with anti-CD3 and anti-CD28 in the presence of 10 ng/ml IL-4 (Peprotech), 10 μg/ml anti-IL-12, and 10 μg/ml anti-IFN-γ, and infected with the above lentiviral vectors, or a control vector pRV-GFP. At day 2, GFP^{+} cells were sorted, and cultured under Th2 condition. At day 7, the cells were restimulated with PMA and ionomycin in the presence of Golgi-stop for 5 hr, and IL-4-producing cells were determined by intracellular staining on a GFP^{+} gate.

T cell isolation and activation

$CD4^+$ T cells were enriched using CD4 T cell enrichment columns (R & D Systems, Minneapolis, MN). Naive $CD4^+CD25^-CD62L^{hi}CD44^{lo}$ T cells were then sorted by flow cytometry from enriched $CD4^+$ T cells. For acute stimulation, the sorted naive $CD4^+$ T cells were activated with IL-4 (5 ng/ml) with or without anti-CD3 and anti-CD28 as previously described (Zhang et al., 2002; Zhang et al., 2003). The cells were lysed in 0.5 % NP-40 lysis buffer or in RIPA buffer (Li et al., 2004; Zhang et al., 2002) where indicated.

In vitro assay for cytokine production

Naïve $CD4^+$ T cells were stimulated with with plate-bound anti-CD3 (2 μg/ml) plus anti CD28 (1 μg/ml) for 48 h, and the supernatants were collected for detection of IL-4, IL-5, IL-9, IL-13, IL17, and IFN-γ by ELISA with ELISA kits (eBiosciences)

In vitro Th1, Th2, Th9, and Th17 differentiation assays

Naïve $CD4^+$ T cells isolated from WT and Cbl-$b^{-/-}$ mice were stimulated with plate-bound anti-CD3 (2 μg/ml) and anti-CD28 (1 μg/ml) in the presence of Th1, Th2, or Th9 cytokine cocktails: IL-12 (5 ng/ml) and anti-IL-4 (10 μg/ml) (for Th1 condition), IL-4 (5 ng/ml), anti-IFN-γ (10 μg/ml), and anti-IL-12 (10 μg/ml) (for Th2 condition), and IL-4 (5 ng/ml), TGF-β (2 ng/ml), and anti-IFN-γ (10 μg/ml) for Th9 condition) for 3 days. Naïve DO11.10.*Cblb*$^{+/+}$ and DO11.10.*Cblb*$^{-/-}$ $CD4^+$ T cells

were activated with irradiated BALB/c T-depleted splenocytes loaded with $OVAp_{323-339}$ peptide (2 μg/ml) in the presence of a Th2 or Th9 cytokine cocktail for 3 days. The cells were collected, washed thoroughly with fresh medium and cultured in the presence of human IL-2 (50 U/ml) and Th1, Th2, or Th9 cytokine cocktails for 2 days. The cells were then collected and restimulated with PMA/ionomycin for 5 h in the presence of Golgi Stop, and the IFN-γ-, IL-4-, and IL-9-producing cells were determined by intracellular staining. For in vitro Th17 differentiation, naïve $CD4^+$ T cells isolated from WT and *Cblb*$^{-/-}$ mice were stimulated with T-depleted splenocytes together with anti-CD3 (5 μg/ml) plus anti-CD28 (2 μg/ml) in the presence of TGF-β (5 ng/ml) plus IL-6 (20 ng/ml) for 4 days. The cells were then collected and restimulated with PMA plus Ionomycin. IL-17-producing cells were determined by intracellular staining.

Asthma Induction

Mice (5 mice/group) were immunized by intraperitoneal (i.p.) injection of OVA on day 0 and day 14. After 21 days, challenge doses of OVA were given through the airways by subjecting mice to 50 ml of a solution of 20 mg/ml OVA induced into aerosol form in a Plexiglas chamber with a nebulizer for 45 min. The challenge was performed once a day for three consecutive days, and methacholine challenge was performed 24 h after the final dose of OVA. Respiratory resistance was measured through a computer-controlled small-animal ventilator (SAV) (Flexivent; SCIREQ). The mice were sacrificed and assessed for allergic

inflammation of the lungs 24 hr after the last aerosol exposure. BAL fluid was collected at the sacrifice, and cytokine concentrations in the BAL fluid were measured by ELISA. Cell differentials in the BAL fluid were assessed as described (Myou et al., 2003). Serum IgE level was measured by ELISA. BALB/c nude mice were adoptively transferred by i.v. injection of naïve WT or *Cblb*$^{-/-}$ CD4^{+} T cells, or WT, *Cblb*$^{-/-}$, *Stat6*$^{-/-}$, and *Cblb*$^{-/-}$*Stat6*$^{-/-}$ CD4^{+} T cells (5 x 10^{6}/mouse) which were permitted to equilibrate 30 days after transfer to avoid the effects of homeostatic proliferation (Qiao et al., 2007). The resulting mice were immunized and challenged with OVA. The cytokine concentrations were measured in BAL fluid. Serum IgE was detected by ELISA. Where specified, mice were intravenously injected with 20 μg control antibody or anti-IL-9 (222622; R&D Systems) 30 min before each challenge (Chang et al. 2010).

In vivo T cell activation upon OVA/alum immunization

To measure in vivo T cell activation upon OVA/alum immunization, WT BLALB/c mice were adoptively transferred with naïve CD4^{+} T cells from DO11.10.*Rag1*$^{-/-}$ and DO11.10.*Rag1*$^{-/-}$*Cblb*$^{-/-}$ mice, and immunized (i.p.) with OVA at 100 μg/ml dose in alum, and the mice were sacrificed each day for five consecutive days to analyze the expression of CD25 and CD69 at the cell surface of KJ1-26^{+} T cells by flow cytometry.

Detection of ubiquitination

For detection of ubiquitination of Stat6, 293T cells were transfected with various constructs; 48 h later, 4 x 10^6 cells were lysed in RIPA buffer. For detection of Stat6 in primary T cells, $CD4^+$ T cells from WT and *Cblb*$^{-/-}$ mice or WT and *Cblb*^{C373A} mice were stimulated with IL-4 in the presence or absence of anti-CD3 plus anti-CD28, lysed, immunoprecipitated with anti-Stat6, and blotted with anti-ubiquitin.

GST pull-down assay

For the GST–Stat6 pull-down assay, 5 ug GST-Stat6, GST-Stat6 TAD, and GST-Stat6 SH2 fusion protein were incubated with 200 µg of BALB/c $CD4^+$ T cell lysates followed by glutathione-sepharose beads. The binding reactions were incubated at room temperature for 2 h. After extensive washing, the bead-bound fractions were probed with the antibodies against Cbl-b, or c-Cbl, Itch, TRAF-2, TRAF-6, and Nedd4 where indicated.

Chromatin immunoprecipitation

$CD4^+$ T cells from WT and *Cblb*$^{-/-}$ mice were stimulated with anti-CD3, anti-CD28, and IL-4, or anti-CD3, anti-CD28, IL-4, and TGF-β for 30 min and 24 h. The stimulated cells were cross-linked with paraformaldehyde and then sonicated. The lysates were subjected to a ChIP assay with anti-Stat6 mAb or control IgG using ChIP assay kits (Millipore) as previously described (Onodera et al., 2010).

Quantitative representations of the results are shown as relative band intensities measured by The Li-Cor Odyssey® Infrared Imaging System (Li-Cor). The specific primers used for the *gata3* locus S7 region: forward, 5'-GCCTCTCTACTGGGCGTCTTCCAG-3', and reverse, 5'-TAGCGAGACCTAGGCTCACTGGTC-3' (Onodera et al., 2010). The specific primers used for the *Il9* promoter, forward, 5'-ACTGATACCCAGTGCCCAC, and reverse, 5'-ACACAGACCTGGGCTTTCA (Yang, et al., 2013).

SUPPLEMENTAL REFERENCES

Chang, H-C., Sehra, S., Goswami, R., Yao, W., Yu, Q., Striesky, G.L., Jabeen, R., McKinley, C., Ahyi, A-N., Han, L., Nguyen, E.T., Robertson, M., Perumal, N.B., Tepper, R.S., Nutt, S.L., and Kaplan, M.H. (2010). The transcription factor PU.1 is required for the development of IL-9-producing T cells and allergic inflammation. Nat. Immunol. *11*, 527-534.

Chiang,Y.J., Kole,H.K., Brown,K., Naramura,M., Fukuhara,S., Hu,R.-J., Jang,I.K., Gutkind,J.S., Shevach,E., and Gu,H. (2000). Cbl-b regulates the CD28 dependence of T-cell activation. Nature *403*, 216-220.

Ko, J-K. and Ma, J. (2005). A rapid and efficient PCR-based mutagenesis method applicable to cell physiology study. Am. J. Physiol. Cell Physiol. *288*:C1273-C1278.

Li,D., Gal,I., Vermes,C., Alegre,M.L., Chong,A.S., Chen,L., Shao,Q., Adarichev,V., Xu,X., Koreny,T., Mikecz,K., Finnegan,A., Glant,T.T., and Zhang,J. (2004). Cutting Edge: Cbl-b: One of the key molecules tuning CD28- and CTLA-4-mediated T cell costimulation. J. Immunol. *173*, 7135-7139.

Myou,S., Leff,A.R., Myo,S., Boetticher,E., Tong,J., Meliton,A.Y., Liu,J., Munoz,N.M., and Zhu,X. (2003). Blockade of inflammation and airway hyperresponsiveness in immune-sensitized mice by dominant-negative phosphoinositide 3-kinase-TAT. J. Exp. Med. *198*, 1573-1582.

Oksvold,M.P., Dagger,S.A., Thien,C.B., and Langdon,W.Y. (2008). The Cbl-b RING finger domain has a limited role in regulating inflammatory cytokine production by IgE-activated mast cells. Mol. Immunol. *45*, 925-936.

Onodera, A., Yamashita, M., Endo, Y., Kuwahara, M., Tofukuji, S., Hosokawa, H., Kanai, A., Suzuki, Y., and Nakayama, T. (2010). STAT6-mediated displacement of polycomb by trithorax complex establishes long-term maintenance of *GATA3* expression in T helper type 2 cells. J. Exp. Med. *207*, 2493-2506.

Qiao,G., Lei,M., Li,Z., Sun,Y., Minto,A., Fu,Y.X., Ying,H., Quigg,R.J., and Zhang,J. (2007). Negative regulation of CD40-mediated B cell responses by E3 ubiquitin ligase Casitas-B-lineage lymphoma protein-B. J. Immunol. *179*, 4473-4479.

Yang, X.O., Zhang, H., Kim, B-S., Niu, X., Peng, J., Chen, Y., Kerketta, R., Lee, Y-H., Chang, S.H., Corry, D.B., Wang, D., Watowich, S.S., and Dong, C. (2013) The signaling suppressor CIS controls proallergic T cell development and allergic airway inflammation. Nat. Immunol. *14*, 732-740.

Zhang,J., Bardos,T., Li,D.-D., Gal,I., Vermes,C., Xu,J.-Y., Mikecz,K., Finnegan,A., Lipkowitz,S., and Glant,T.T. (2002). Cutting Edge:Regulation of T

cell activation threshold by CD28 costimulation by targeting Cbl-b for ubiquitination. J. Immunol. *169*, 2236-2240.

Zhang,J., Bárdos,T., Shao,Q., Tschopp,J., .Mikecz,K., Glant, and Finnegan,A. (2003). IL-4 potentiates activated T cell apoptosis via an IL-2-dependent mechanism. J. Immunol. 1*70,* 3495-3503.
